# Different Chitin Synthase Genes Are Required for Various Developmental and Plant Infection Processes in the Rice Blast Fungus *Magnaporthe oryzae*

**DOI:** 10.1371/journal.ppat.1002526

**Published:** 2012-02-09

**Authors:** Ling-An Kong, Jun Yang, Guo-Tian Li, Lin-Lu Qi, Yu-Jun Zhang, Chen-Fang Wang, Wen-Sheng Zhao, Jin-Rong Xu, You-Liang Peng

**Affiliations:** 1 State Key Laboratory of Agrobiotechnology and MOA Key Laboratory of Plant Pathology, China Agricultural University, Beijing, China; 2 Purdue-NWAFU Joint Research Center and State Key Laboratory of Crop Stress Biology for Arid Areas, College of Plant Protection, Northwest A&F University, Yangling, Shaanxi, China; 3 College of Plant Protection, Northwest A&F University, Yangling, Shaanxi, China; University of Melbourne, Australia

## Abstract

Chitin is a major component of fungal cell wall and is synthesized by chitin synthases (Chs). Plant pathogenic fungi normally have multiple chitin synthase genes. To determine their roles in development and pathogenesis, we functionally characterized all seven *CHS* genes in *Magnaporthe oryzae*. Three of them, *CHS1*, *CHS6*, and *CHS7*, were found to be important for plant infection. While the *chs6* mutant was non-pathogenic, the *chs1* and *chs7* mutants were significantly reduced in virulence. *CHS1* plays a specific role in conidiogenesis, an essential step for natural infection cycle. Most of *chs1* conidia had no septum and spore tip mucilage. The *chs6* mutant was reduced in hyphal growth and conidiation. It failed to penetrate and grow invasively in plant cells. The two MMD-containing chitin synthase genes, *CHS5* and *CHS6*, have a similar expression pattern. Although deletion of *CHS5* had no detectable phenotype, the *chs5 chs6* double mutant had more severe defects than the *chs6* mutant, indicating that they may have overlapping functions in maintaining polarized growth in vegetative and invasive hyphae. Unlike the other *CHS* genes, *CHS7* has a unique function in appressorium formation. Although it was blocked in appressorium formation by germ tubes on artificial hydrophobic surfaces, the *chs7* mutant still produced melanized appressoria by hyphal tips or on plant surfaces, indicating that chitin synthase genes have distinct impacts on appressorium formation by hyphal tip and germ tube. The *chs7* mutant also was defective in appressorium penetration and invasive growth. Overall, our results indicate that individual *CHS* genes play diverse roles in hyphal growth, conidiogenesis, appressorium development, and pathogenesis in *M. oryzae*, and provided potential new leads in the control of this devastating pathogen by targeting specific chitin synthases.

## Author Summary

Chitin is one of the major components of cell wall that plays vital roles in hyphal tip growth and fungal morphogenesis. Biosynthesis of chitin is catalyzed by chitin synthases, a well-known fungicide target. However, systematic characterization of chitin synthase genes has not yet been reported in plant pathogenic ascomycetes. To determine their roles in development and pathogenesis, in this study we functionally characterized all the seven chitin synthase genes (*CHS1*-*CHS7*) in *Magnaporthe oryzae*, a model for studying fungal development and pathogenesis. While *CHS2*, *CHS3*, *CHS4*, and *CHS5* are dispensable for plant infection, *CHS6* is essential for pathogenesis. The *chs6* mutant failed to penetrate plant cells and develop infectious hyphae. Two other chitin synthase genes, *CHS1* and *CHS7*, also are important for virulence. The *chs1* and *chs7* mutants caused only rare lesions on rice seedlings. Other than plant infection, *CHS1* and *CHS7* also play specific roles during conidiogenesis and appressorium formation, respectively. Interestingly, the *chs7* mutant was blocked in appressorium formation by germ tubes on artificial hydrophobic surfaces but was normal by hyphal tips and on plant surfaces. Different chitin synthase genes may be involved during appressorium formation by different fungal tissues or on different surfaces in *M. oryzae*.

## Introduction

Chitin, a microfibrillar β-1,4-linked homopolymer of *N*-acetyl-glucosamine (GlcNAc), is one of the major structural components of the fungal cell wall [1]. Chitin synthases are key enzymes catalyzing the polymerization of GlcNAc [1]. They are usually localized to cytoplasmic membrane and have attracted considerable attentions as targets for developing fungicides [2], [3]. Chitin synthase (*CHS*) genes from various fungi have been grouped into seven classes [4]. All chitin synthases have chitin synthase domains and transmembrane domains in common. In addition, the class V and class VI chitin synthases contain the myosin motor domain (MMD) at their N-terminal [5], [6]. Myosins are known as mechanoenzymes that convert chemical energy released by ATP hydrolysis into a mechanical force that is directed along actin filaments [7].

Fungi are different in chitin contents and in the composition of chitin synthases. In the budding yeast *Saccharomyces cerevisiae*, chitin constitutes 1–2% of the total dry weight, and is a minor cell wall component and mainly exists at the mother-daughter cell junction and septum [8]. *S. cerevisiae* has three *CHS* genes with distinct functions in cell wall expansion, septum formation, and budding [9]–[11]. Chs1 repairs the weakened cell wall of daughter cells after separation. Chs2 synthesizes chitin in primary septa. It is essential for both septum formation and cell division [12]. Chs3 chitin synthase is required for chitin ring formation at the base of emerging buds and chitin synthesis in the lateral cell. Chs3 is responsible for 90% of chitin synthesis while Chs1 and Chs2 are involved in only small amounts of chitin synthesis at extreme parts of cells [13]. Unlike the budding yeast, *Schizosaccharomyces pombe* has only two chitin synthase genes [14]. *Ashbya gossybii* and *Candida albicans* have three and four *CHS* genes, respectively [15].

In filamentous fungi, chitin content has been reported to reach up to 10–20% [16]. Filamentous ascomycetes generally have seven or eight *CHS* genes, which may reflect their greater complexity of growth and development and higher chitin content than the ascomycetous yeasts. A number of *CHS* genes have been characterized in the model filamentous ascomycetous fungi *Neurospora crassa* and *Aspergillus nidulans*. *N. crassa* has seven *CHS* genes, one for each class [17]. *CHS1* (I) plays a major role in cell wall biogenesis [18]. The *chs-1* ^RIP^ mutant had abnormal branching, swollen hyphal tips, reduced growth rate, and increased sensitivity to Nikkomycin Z [18]. Morphologically, the *chs-2* ^RIP^ mutant was indistinguishable from the wild type. However, it had an increased sensitivity to the phosphatidylcholine biosynthesis inhibitor edifenphos [19]. The *chs-4* ^RIP^ mutant did not show any reduction in chitin content under standard growth conditions. However, chitin enrichment in the cell wall induced by sorbose treatment was impaired in the *chs-4* ^RIP^ [20]. In transformants expressing the *CHS3-GFP* or *CHS6-GFP* constructs, GFP signals were mainly detected in the hyphal tip, suggesting that they may be invovled in polarized growth [21]. *A. nidulans* has eight *CHS* genes [3]. One of the two class I *CHS* genes, *chsB*, functions in the formation of normal cell wall in vegetative hyphae, conidiophores, and developing conidia [22], [23]. The *chsB* mutant has similar defects in hyphal growth with the *chs-1* ^RIP^ mutant [18]. The *csmA* and *csmB* genes encode class VI and V chitin synthases, respectively. The growth rate of the *csmA* mutant was similar to that of the wild type, but it was defective in cell wall integrity and tended to produce swollen hyphal tips and intrahyphal hyphae [24]. The *csmB* mutant had similar phenotypes with the *csmA* mutant and deletion of both *csmA* and *csmB* appeared to be lethal [6], suggesting that these two *CHS* genes had overlapping functions in *A. nidulans*. Although the *chsA* (II), *chsC* (III), and *chsD* (IV) mutants have no obvious phenotypes, the *chsA chsD* double mutant was reduced in conidiation and the *chsA chsC* double mutant was defective in hyphal growth and sexual/asexual development [25]–[27]. *A. fumigatus* also has eight chitin synthase genes. In general, the *A. fumigatus CHS* genes have similar functions as their orthologs in *A. nidulans* [3], [28].

The corn smut fungus *Ustilago maydis*, a basidiomycete, has two each for class II (*CHS3*, and *CHS4*), and class IV (*CHS5* and *CHS7*), and one each for class I (*CHS1*), class III (*CHS2*), class V (*CHS6*), and class VI (*MCS1*) chitin synthase genes [5]. Deletion of the *chs1* gene has no obvious effects on mating, virulence and dimorphic behavior, but *chs1* cells are slightly shorter [5], [29]. The *chs2* mutant has no obvious effects on mating, virulence, or dimorphism but the *chs3* and *chs4* mutants produced fewer hyphae on medium with synthetic pheromone [5], [29]. The *CHS5*, *CHS6*, *CHS7*, and *MCS1* genes are all important for virulence [5], [30], [31]. While the *chs5* mutant forms irregular yeast cells, the *chs7* mutant has a cell separation defect. The *chs6* and *mcs1* mutants have thicker cell wall than the wild type. The *mcs1* mutant has no morphological defects *in vitro* but forms large aggregates of spherical cells.

In plant pathogenic ascomycetes, to date there is no report on systematic characterization of *CHS* genes although a number of chitin synthase genes have been shown to be important for virulence in various plant pathogenic fungi, including *BcCHS1* and *BcCHSIIIa* of *Botrytis cinerea* [32], [33], *GzCHS5* and *GzCHS7* of *Fusarium graminearum* [34], *CHS5* and *CHS7* of *F. verticillioides* [35], and *CgCHSV* of *Colletotrichum graminicola* [36]. In the vascular wilt pathogen *F. oxysporum*, four of its nine *CHS* genes have been characterized. The *CHS1* (III) gene is not important for plant infection but the *chs2* (II) mutant is reduced in virulence on tomato plants. Moreover, the stress conditions affected normal development in the *chs2* mutant but not in the *chs1* mutant [37]. The *CHSV* (V) and *CHSVb* (VI) are two MMD *CHS* genes. Deletion of the *CHSV* or *CHSVb* gene resulted in a significant reduction in virulence and production of swollen, balloon-like structures along the hyphae [38]. The *chsV chsVb* double mutant was viable but failed to infect and colonize tomato plants [39].

In the genome of the rice blast fungus *Magnaporthe oryzae* [40], there are seven predicted *CHS* genes, including *CHS7* [41]. This study was initiated when the genome sequence of *M. oryzae* was first published [40]. We functionally characterized all seven *CHS* genes that were named after their *N. crassa* orthologs. Three of them, *CHS1*, *CHS6*, and *CHS7*, were important for plant infection. The *chs1* mutant had altered conidium morphology and was defective in conidium germination. It was reduced in invasive growth and virulence. The *chs6* mutant was non-pathogenic. Appressoria formed by the *chs6* mutant were defective in penetration and invasive hyphae in plant cells. Germ tubes of the *chs7* mutant failed to form appressoria on artificial hydrophobic surfaces. However, it was normal in appressorium formation on plant surfaces or by hyphal tips, indicating that defects of the *chs7* mutant in appressorium formation are surface- and cell type-specific. Appressorium formation by hyphal tips and germ tubes may involve different chitin synthase genes in *M. oryzae*. In addition, we generated and characterized *chs5 chs6* double mutants to determine the relationship among these two chitin synthase genes with similar structures and expression profiles. Results from this study indicate that individual *CHS* gene plays diverse roles in hyphal growth, conidiogenesis, appressorium development, and pathogenesis in *M. oryzae*, and provided potential new leads in the control of this devastating pathogen by targeting specific chitin synthases.

## Results

### Seven chitin synthase genes of *M. oryzae* are differentially expressed

**Figure 1:**
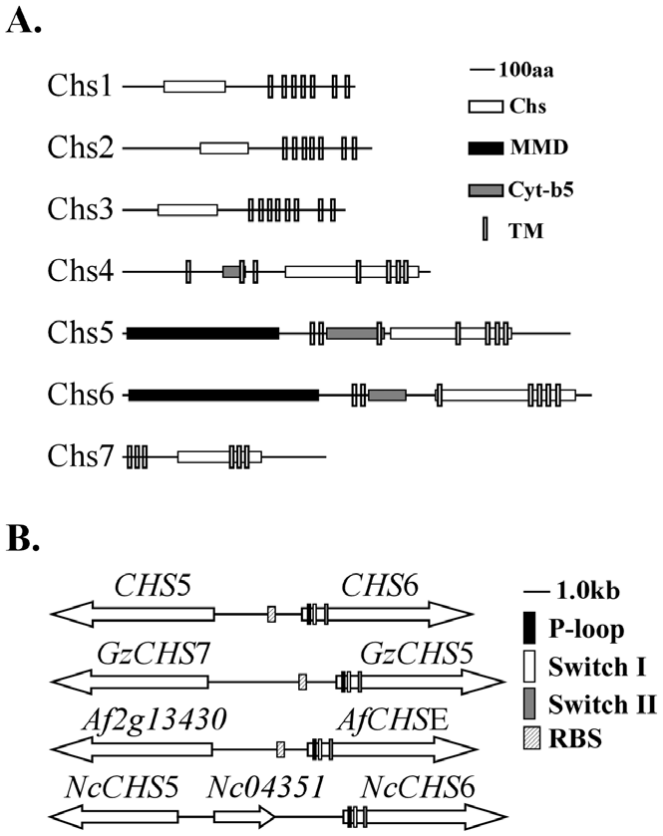
The seven predicted *CHS* genes in *M. oryzae*. (A) Domain structures of the seven chitin synthases in *M. oryzae*. Chs, chitin synthase domain; Cyt-b5, cytochrome b5-like heme/steroid binding domain; MMD, myosin motor domain; TM, transmembrane domain. (B) Directions and chromosomal positions of *CHS5* and *CHS6* in *M. oryzae* and their orthologs in *F. graminearum*, *A. fumigatus*, and *N. crassa*. RBS, Rlm1p-binding sequence.

The *M. oryzae* genome contains seven predicted chitin synthase genes MGG_01802.6, MGG_04145.6, MGG_09551.6, MGG_09962.6, MGG_13014.6, MGG_13013.6, and MGG_06064.6 (www.broad.mit.edu/annotation/genome/magnaporthe_grisea). They are named as *CHS1-CHS7* (Figure S1) according to their orthologs in *N. crassa* [17]. In addition to the core chitin synthase domain (Chs, IPR004835), all the *M. oryzae* chitin synthase genes have multiple transmembrane (TM) domains (Figure 1A). *CHS4*, *CHS5*, and *CHS6* also have one cytochrome b5-like heme/steroid binding domain (Cyt-b5, IPR001199) upstream from the Chs domain. Structurally, *CHS5* and *CHS6* are more similar to each other than to other *CHS* genes in *M. oryzae* (Figure 1A). Both of them have an N-terminal myosin motor domain (MMD, IPR001609) and they are closely linked in the genome (Figure 1B).

**Figure 2:**
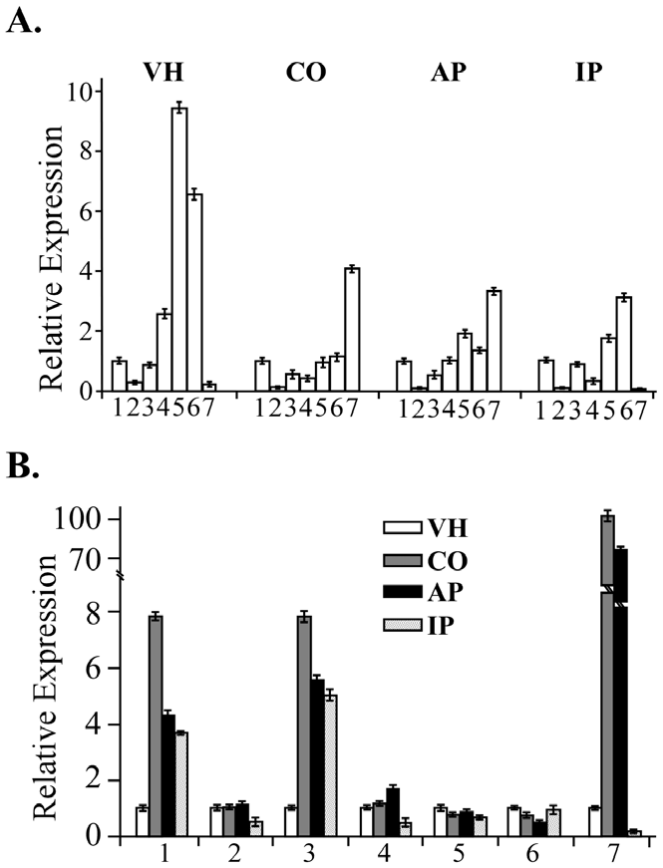
Expression profiles of seven *CHS* genes assayed by qRT-PCR. RNA samples of the wild-type strain P131 were isolated from vegetative hyphae grown in minimal medium (VH), conidia harvested from 10-day-old oatmeal agar cultures (CO), 24 h appressoria (AP), and infected plant leaves (IP). The relative expression level of individual *CHS* genes was analyzed with the 2^−*ΔΔ*Ct^ method with the actin gene as the internal control for normalization. (A) Comparison of the transcript abundance of seven *CHS* genes in VH, CO, AP, and IP. The expression level of *CHS1* was arbitrarily set to 1. Columns 1 to 7 represent the chitin synthase genes *CHS1* to *CHS7*. (B) Comparison of the transcript abundance of individual *CHS* genes in four different fungal tissues. The expression level of each *CHS* gene in vegetative hyphae (VH) was arbitrarily set to 1. Mean and standard errors were determined with data from three independent replicates.

The expression levels of the *CHS* genes in vegetative hyphae, conidia, appressoria, and infected rice leaves were assayed by quantitative RT-PCR (qRT-PCR). In comparison with other *CHS* genes, the abundance of *CHS2* transcripts was relatively low in all four fungal tissues (Figure 2A). The *CHS7* gene had the highest expression level in conidia and appressoria but the lowest expression level in vegetative hyphae and infected rice leaves among all seven *CHS* genes (Figure 2A). In contrast, both *CHS5* and *CHS6* had higher expression levels in vegetative hyphae and infected rice leaves than other *CHS* genes (Figure 2A), suggesting that they played more important roles in the vegetative or invasive hyphal growth than other *CHS* genes.

The *CHS1* and *CHS3* genes had similar expression profiles, with the lowest expression level in vegetative hyphae and the highest in conidia (Figure 2B). Their expression levels also were increased in appressoria and infected rice leaves in comparison with vegetative hyphae. *CHS7* is known to be important for appressorium formation on artificial surfaces [41]. However, its expression level was higher in conidia than in appressoria (Figure 2B). The open reading frames (ORFs) of *CHS5* and *CHS6* are only 2,957-bp apart. Their orthologs are also located next to each other in *F. oxysporum* [39], *A. nidulans* [6], *A. fumigatus*, and *F. graminearum* (Figure 1B). Similar expression profiles of *CHS5* and *CHS6* indicate that they may have some common promoter elements due to their head-to-head arrangement on the chromosome (Figure 1B). As expected, one putative Rlm1p-binding sequence (RBS) CTAcgcaTAG was found at 917-bp upstream of the *CHS6* ORF. Putative RBS also exists in the promoter regions of the *CHS5* and *CHS6* orthologs in *F. graminearum* and *A. fumigatus* (Figure 1B).

### Generation of mutants deleted of individual *CHS* genes

**Figure 3:**
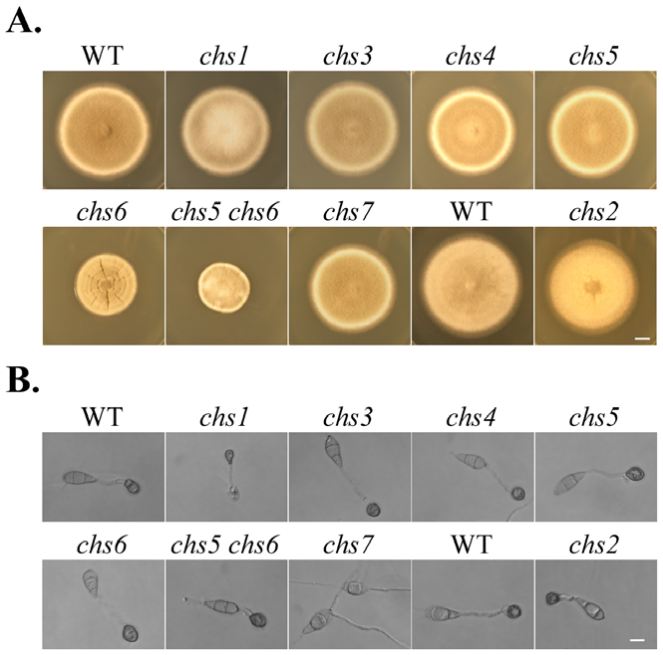
Colony morphology and melanized appressoria of the *chs* deletion mutants. (A) Colonies of the wild-type strains P131 and S1528 and the *chs1-chs7* mutants formed on the complete medium (CM) at 25°C. Photos were taken at 7 days post-inoculation. Bar = 10 mm. (B) Appressorium formation assays with conidia from the same set of the wild-type and mutant strains. Representative images were taken after 24 h incubation on glass coverslips. Germ tubes of the *chs7* mutant failed to form appressoria. Bar = 10 µm.

In order to systematically characterize their biological functions in *M. oryzae*, mutants deleted of individual *CHS* genes were generated by the conventional gene replacement approach (Table 1; Table S1). For each gene, at least two independent null mutants with identical phenotypes were generated (Table 1). All the *chs* mutants were identified by PCR and confirmed by Southern blot analyses (Figure S2). Although the growth rate or colony morphology was altered in some mutants, none of the *CHS* genes were essential for vegetative growth. While deletion of *CHS2* and *CHS6* resulted in a 20% and 31% reduction, respectively, in the growth rate, all the other *chs* mutants had no significant changes in vegetative growth (Table 2). In comparison with the wild type, all the *chs* mutants had normal colony surface hydrophobicity (Figure S3). However, unlike other *chs* mutant, the *chs6* mutant formed colonies with short, compact aerial hyphae and wrinkled surface on CM plates (Figure 3A). In contrast, the *chs1* mutant tended to produce fluffy colonies and was slightly reduced in the melanization of aerial hyphae (Figure 3A).

**Table 1:** Fungal strains used in this study.

Strain	Brief description	Reference
P131	Wild type, *MAT1-1*	[55]
S1528	Wild type, *MAT1-2*	[53]
LA11	*chs1* mutant of P131	this study
LA40	*chs1* mutant of P131	this study
LA1	*chs3* mutant of P131	this study
LA3	*chs4* mutant of P131	this study
LA28	*chs4* mutant of P131	this study
LA8	*chs5* mutant of P131	this study
LA14	*chs6* mutant of P131	this study
LA26	*chs6* mutant of P131	this study
LA12	*chs7* mutant of P131	this study
LA2	*chs2* mutant of S1528	this study
LA6	*chs2* mutant of S1528	this study
LA51	*chs3* mutant of S1528	this study
LA53	*chs3* mutant of S1528	this study
LA41	*chs5* mutant of S1528	this study
LA43	*chs5* mutant of S1528	this study
LA49	*chs5 chs6* double mutant of P131	this study
LA66	*chs5 chs6* double mutant of P131	this study
LA33	Transformant of LA40 expressing *CHS1-eGFP*	this study
LA71	Transformant of LA12 expressing *CHS7-eGFP*	this study

**Table 2:** Phenotype characterization of *chs* mutants of *M. oryzae*.

Strain	Growth rate (mm/day)^a^	Germination (%)^b^	Conidiation (10^5^spores/plate)^c^	Appressoria formation (%)^d^	Disease index^e^
P131 (WT)	3.4±0.1**^α^** *	95.5±1.9**^α^**	76.9±3.8**^α^**	96.2±0.1**^α^**	62±4**^α^**
LA40 (*chs1*)	3.1±0.1**^α^**	64.3±1.9**^α^**	14.4±0.9**^β^**	61.0±4.1**^β^**	2±1**^β^**
LA1 (*chs3*)	3.2±0.1**^α^**	98.4±1.3**^α^**	73.6±0.7**^α^**	97.4±0.3**^α^**	53±9**^α^**
LA28 (*chs4*)	3.1±0.1**^α^**	98.1±2.9**^α^**	74.4±7.1**^α^**	95.4±1.9**^α^**	60±5**^α^**
LA8 (*chs5*)	3.2±0.1**^α^**	94.8±4.5**^α^**	74.9±3.6**^α^**	96.2±2.3**^α^**	61±3**^α^**
LA26 (*chs6*)	2.1±0.1**^β^**	93.9±1.3**^α^**	5.6±0.2**^β^**	91.5±0.5**^α^**	0±0**^β^**
LA49 (*chs5 chs6*)	1.7±0.1**^β^**	90.4±1.2**^α^**	0.5±0.1**^γ^**	85.2±1.0**^α^**	0±0**^β^**
LA12 (*chs7*)	3.1±0.1**^α^**	95.1±1.8**^α^**	74.2±4.9**^α^**	0.0±0.0**^β^**	10±6**^β^**
S1528 (WT)	3.5±0.1**^α^**	95.6±1.9**^α^**	25.3±2.1**^α^**	97.6±2.0**^α^**	56±10**^α^**
LA6 (*chs2*)	2.9±0.1**^β^**	98.1±1.5**^α^**	4.3±0.3**^β^**	90.6±1.7**^α^**	61±2**^α^**

a Average daily extension in the diameter of complete medium (CM) cultures. Means and standard deviations were calculated from three independent experiments.

b Percentage of conidia germinated after 1 h incubation on hydrophobic surfaces.

c Conidia produced by 14-day-old oatmeal agar plates (Φ 9 cm).

d Percentage of germ tubes formed appressoria after incubation for 20 h on hydrophobic surfaces.

e The number of lesions formed on the 5 cm tip region of infected leaves.

*Data from three replicates were analyzed with two sample t-test. The same greek letter indicated that there was no significant difference with the wild-type or single mutant. Different letters were used to mark statically significant difference (P = 0.01).

Except for the *chs1* mutant, all the other *chs* mutants produced three-celled pyriform conidia with normal morphology. However, conidiation was reduced over 5-fold in the *chs2* and *chs6* mutants (Table 2). The *chs1* mutant tended to form pear-shaped, single-celled conidia. It also was reduced in conidiation but to a less degree than the *chs2* and *chs6* mutants (Table 2). When tested with different stresses, we found that the *chs6* mutant but not the other *chs* mutants had increased susceptibility to osmotic and cell wall stresses (Table 3). Its growth rate on CM with 0.7 M NaCl was reduced over 50% in comparison with that on regular CM (Table 3).

**Table 3:** Assay for inhibitory effects of various chemicals on vegetative growth.

	0.7 M NaCl	1.2 M Sorbitol	5 mM H_2_O_2_	50 µg/ml CFW	50 µg/ml CR	5 mM NZ
P131 (WT)	37.9±2.0**^α^**	34.6±0.8**^α^**	8.0±1.1**^α^**	6.4±1.2**^α^**	7.2±2.0**^α^**	3.2±1.2**^α^**
LA40 (*chs1*)	40.9±0.7**^α^**	35.2±1.1**^α^**	13.1±1.1**^β^**	4.1±2.5**^α^**	5.7±1.2**^α^**	4.9±2.1**^α^**
LA1 (*chs3*)	40.6±1.3**^α^**	33.3±1.2**^α^**	6.5±1.2**^α^**	8.1±1.2**^α^**	20.3±1.2**^β^**	5.6±1.2**^α^**
LA28 (*chs4*)	38.9±0.7**^α^**	38.9±0.7**^α^**	11.8±1.2**^α^**	14.4±1.2**^β^**	6.7±2.5**^α^**	7.6±2.1**^α^**
LA8 (*chs5*)	38.2±1.2**^α^**	33.3±1.2**^α^**	5.6±1.2**^α^**	4.8±0.0**^α^**	6.5±1.2**^α^**	1.6±1.2**^α^**
LA26 (*chs6*)	52.3±3.5**^β^**	41.5±1.0**^β^**	6.1±2.2**^α^**	32.3±0.7**^β^**	36.9±3.4**^β^**	23.0±3.6**^β^**
LA49 (*chs5chs6*)	34.0±5.1**^α^**	6.4±1.9**^γ^**	13.5±5.8**^β^**	31.9±4.2**^β^**	36.3±3**^β^**	N/A
LA12 (*chs7*)	37.5±0.0**^α^**	34.1±1.2**^α^**	7.5±0.0**^α^**	9.1±1.2**^α^**	7.5±0.0**^α^**	7.5±0.0**^α^**
S1528 (WT)	36.9±1.6**^α^**	45.3±1.0**^α^**	2.3±0.0**^α^**	12.2±1.1**^α^**	1.5±0.4**^α^**	5.3±1.2**^α^**
LA6 (*chs2*)	39.7±8.0**^α^**	55.9±0.7**^β^**	8.4±1.2**^α^**	38.1±4.4**^β^**	9.3±1.0**^α^**	12.6±1.2**^β^**

The inhibition extent (%) = ((the diameter of regular CM cultures-the diameter of cultures on CM with different chemical)/the diameter of regular CM cultures)×100; CFW, Calcofluor white; CR, Congo red; NZ, Nikkomycin Z. N/A, not assayed. Data from three replicates were analyzed with two sample t-test. The same greek letter indicated that there was no significant difference with the wild-type or single mutant. Different letters were used to mark statically significant difference (P = 0.01).

### Chitin contents and chitin synthase activities in the *chs* mutants

**Figure 4:**
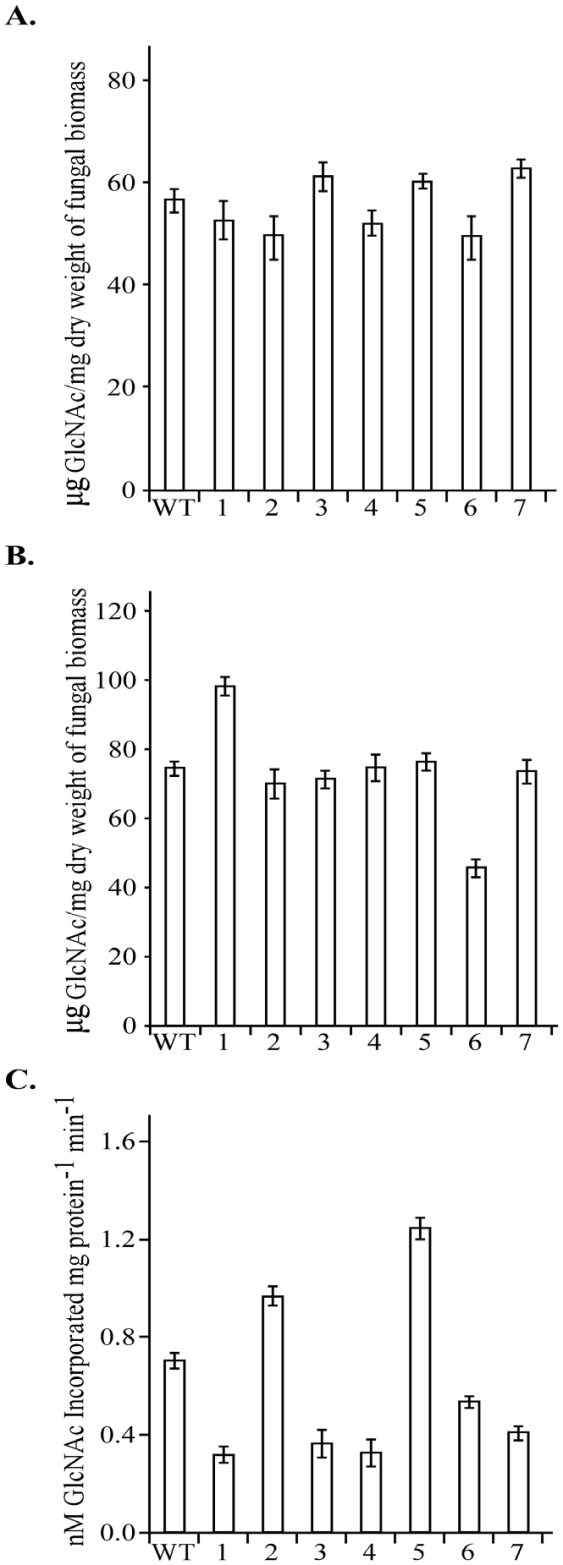
Chitin contents and chitin synthase activities in the wild-type and *chs* mutant strains. The chitin content was assayed with vegetative hyphae harvested from 2-day-old CM cultures (A) or conidia harvested from 10-day-old OTA plates (B) of the wild type and *chs* mutants. The chitin content is expressed in µg of glucosamine hydrochloride per mg of dry-weight of fungal biomass. (C) Chitin synthase activities (nM UDP-N-acetyl-glucosamine incorporated into chitin per mg protein per minute) were assayed with microsomal fractions of proteins isolated from vegetative hyphae of the wild-type and *chs* mutant strains. Mean and standard deviation were calculated with results from three biological replicates.

We then measured the chitin contents in vegetative hyphae and conidia of the *chs* mutants. In comparison with the wild type, none of the *chs* mutants (*chs1*-*chs7*) had significant changes in the chitin content in vegetative hyphae (Figure 4A). However, the chitin content was increased approximately 30% in the *chs1* mutant but reduced approximately 40% in the *chs6* mutant in conidia (Figure 4B). The other *chs* mutants had no obvious changes in the chitin content in conidia.

Chitin synthase activities were assayed with the microsomal fraction extracts from vegetative hyphae of the wild type and *chs* mutants. In comparison with the wild type, the *chs1*, *chs3*, *chs4*, and *chs7* mutants had approximately 2-fold reduction in chitin synthesis activity (Figure 4C). The chitin synthase activity also was reduced in the *chs6* mutant but the reduction was less than 2-fold. In contrast, the *chs*2 and *chs*5 mutants had higher chitin synthase activity than the wild-type strain (Figure 4C). Nevertheless, their increase in chitin synthase activity was less than 2-fold in comparison with the wild type.

### Expression levels of individual *CHS* genes in the *chs* mutants

**Figure 5:**
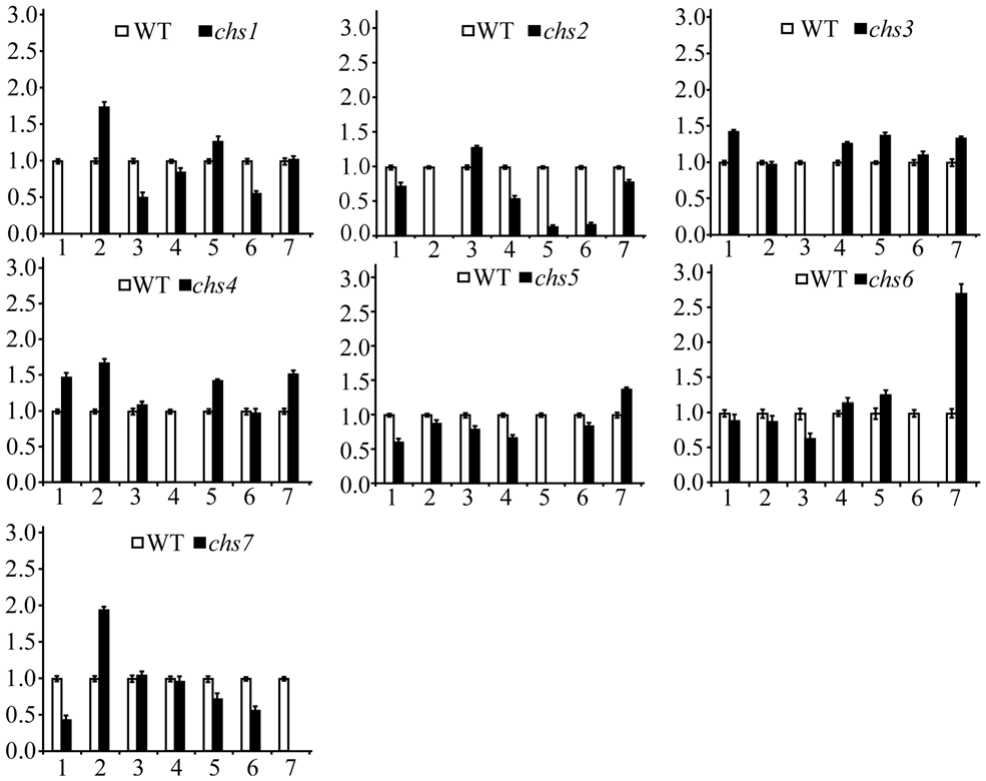
The expression levels of individual *CHS* genes in the wild type and *chs* mutants. RNA samples isolated from vegetative hyphae harvested from 2-day-old CM cultures were used for qRT-PCR assays. The actin gene was used as the endogenous control for normalization. Relative expression levels were estimated with the 2^−ΔΔCt^ method. The expression level of each *CHS* gene in the wild type was arbitrarily set to 1. Mean and standard errors were determined with data from three independent replicates.

The expression levels of individual *CHS* genes were assayed by qRT-PCR with RNA isolated from vegetative hyphae of the wild-type and *chs* mutant strains (Figure 5). In the *chs1* mutant, *CHS2* was increased 1.7-fold, and changes in expression levels in other *CHS* genes were less significantly than that of *CHS2*. In the *chs2* mutant, the expression levels of *CHS5* and *CHS6* were reduced over 5-fold. Although *CHS5* and *CHS6* are closely linked, deletion of any one of these two genes had no significant effects on the expression level of the other one. Among all the *CHS* genes, only the expression level of *CHS7* was increased 2.7-fold in the *chs6* mutant. The *chs7* mutant had 2.2-fold reduced expression of *CHS1* and 1.9-fold increased expression of *CHS2*, respectively. These results indicated that deletion of one specific *CHS* gene had different impacts on the expression of other *CHS* genes during vegetative growth.

### The *CHS1* and *CHS7* genes play important roles in appressorium formation

**Figure 6:**
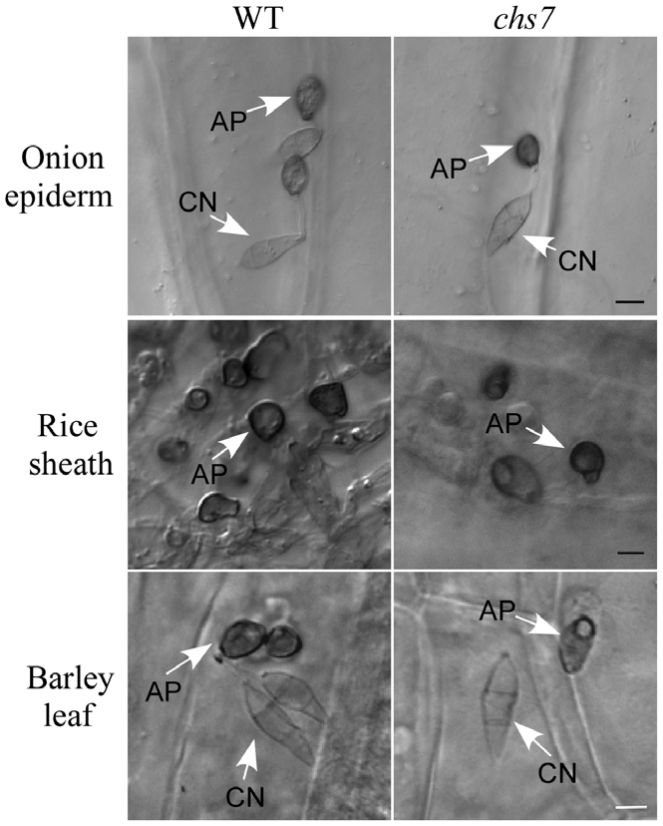
Appressorium formation assays with the *chs7* mutant. Melanized appressoria were formed by the germ tubes of the wild-type strain P131 and *chs7* mutant on onion epidermis cells, rice leaf sheath, and barley leaves. AP, appressoria formed by germ tubes; CO, conidia. Bar = 10 µm.

**Figure 7:**
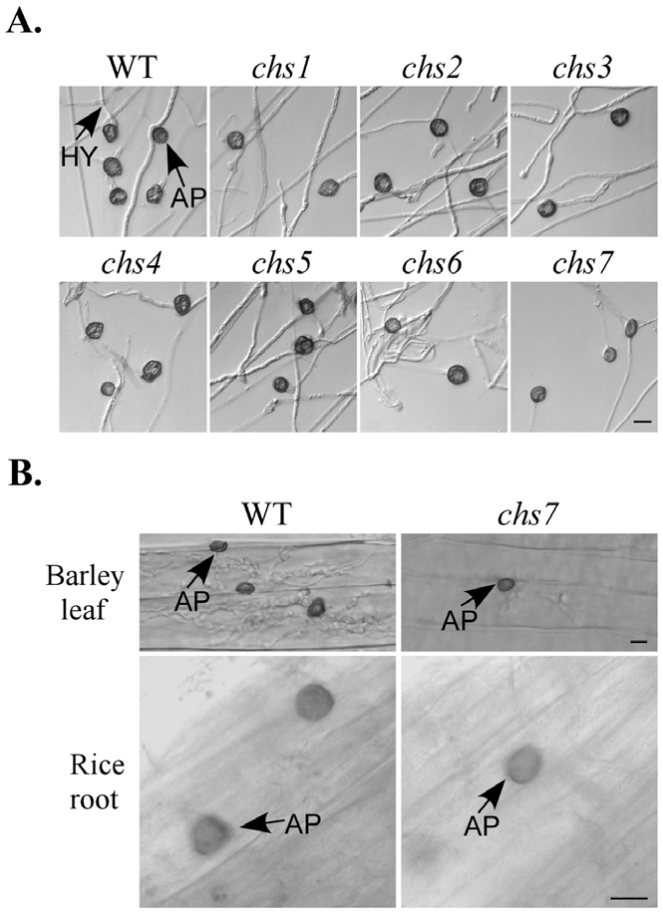
Appressorium formation assays with hyphal tips. (A) Appressoria formed by hyphal tips of the wild-type strain P131 and *chs1*-*chs7* mutants on glass cover slips. Bar = 10 µm. (B) Appressoria formed by hyphal tips of P131 and *chs7* mutant on barley leaf and rice root surfaces. AP, appressoria formed by hyphal tips; HY, hyphae. Bar = 10 µm.

On artificial hydrophobic surfaces, all but the *chs1* and *chs7* mutants were normal in conidium germination and produced melanized appressoria (Table 2). The *chs1* mutant was reduced 33% in conidium germination and only 61% of the germ tubes formed melanized appressoria. Appressoria formed by the *chs1* mutant tended to be smaller than those of the wild type (Figure 3B). Germ tubes of the *chs7* mutant failed to form melanized appressoria on artificial hydrophobic surfaces (Figure 3B), which was consistent with the previous report [41]. However, when the *chs7* mutant was assayed for appressorium formation on plant surfaces, melanized appressoria were observed. Germ tubes of the *chs7* mutant produced appressoria on onion epidermis, rice leaf sheaths, and barley leaves (Figure 6; Table S2), suggesting that its defects in appressorium formation is surface-dependent. Interestingly, like other *chs* mutants, hyphal tips of the *chs7* mutant formed melanized appressoria on artificial hydrophobic surfaces (Figure 7A). Hyphal tips of the *chs7* mutant also formed melanized appressoria on plant surfaces assayed (Figure 7B).

We then assayed the effects of 1,16-hexadecanediol on appressorium formation on artificial surfaces. Germ tubes of the wild type and *chs7* mutant had similar appressoria formation efficiency in the presence of the cutin monomer on hydrophobic surfaces (Table S2), which is consistent with the previous report [41]. Similar to the wild type, cutin monomer treatment had no significant effect on appressorium formation by hyphal tips in the *chs7* mutant (Table S2). These data suggested that appressoria formation by germ tubes and hyphal tips were regulated differently in the *chs7* mutant, and defects of the *chs7* mutant in appressorium formation appeared to be specific to its germ tubes. It is possible that chitin synthase genes have distinct impacts on appressorium formation by germ tubes and hyphal tips.

### The *CHS1*, *CHS6*, and *CHS7* genes are important for plant infection

**Figure 8:**
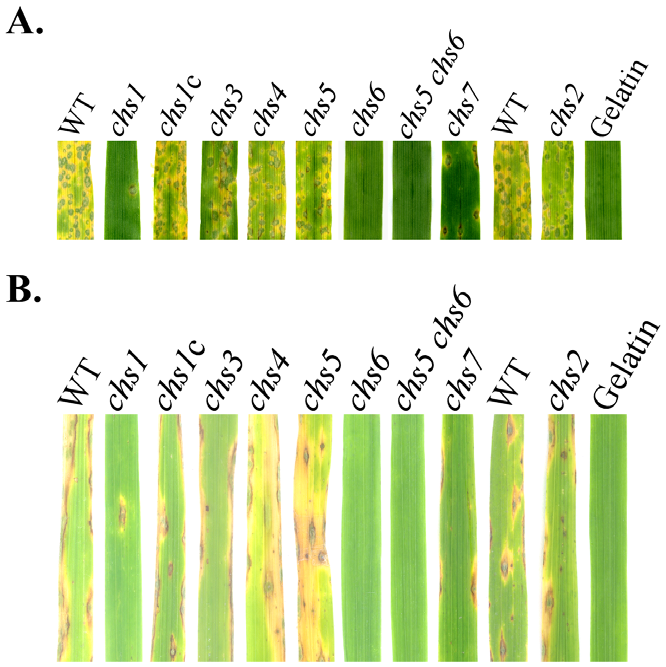
Infection assays with rice and barley seedlings. Conidia were harvested from 10-day-old oatmeal agar cultures of wild-type strains P131 and S1528, and the *chs1-chs7* mutants. (A) Eight-day-old seedlings of barley cultivar Golden Promise were spray inoculated. Typical leaves were photographed 5 dpi. (B) Two-week-old seedlings of rice cultivar LTH were sprayed with conidium suspensions of the wild-type and *chs* mutant strains or 0.25% gelatin solution as the control. Photos were taken at 7 dpi.

When conidia of the chitin synthase mutants were used to spray barley seedlings, the *chs2*, *chs3*, *chs4*, and *chs5* mutants had no significant changes in virulence (Figure 8A; Table 2). The *chs7* mutant was reduced in virulence, which is consistent with the earlier report [41]. However, the *chs1* mutant was more significantly reduced in virulence than the *chs7* mutant. On leaves sprayed with the *chs1* mutant, only rare lesions were observed (Figure 8A). Unlike the other *chs* mutant, the *chs6* mutant was non-pathogenic. On barley leaves sprayed with the *chs6* mutant, no lesions were observed 5 days post inocultion (dpi) (Figure 8A; Table 2). Under the same conditions, numerous blast lesions were observed on leaves inoculated with the wild-type strain P131 (Figure 8A). Even on wounded barley leaves, the *chs6* mutant failed to form any lesions.

We then repeated infection assays with rice seedlings. Similar results were obtained for all the *chs* mutants (Figure 8B). The *CHS2*, *CHS3*, *CHS4*, and *CHS5* appeared to be dispensable for plant infection. In contract, the *CHS6* gene is essential for pathogenesis in *M. oryzae*. The *chs6* mutant failed to cause any lesion. On rice leaves sprayed with the *chs1* and *chs7* mutants, fewer blast lesions were observed than those sprayed with the wild type (Figure 8B).

### *CHS7* is important for appressorium penetration and invasive growth in penetration assays with rice leaf sheaths and barley leaves

**Figure 9:**
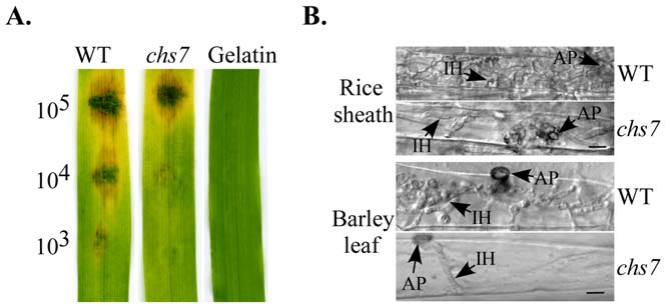
The *chs7* mutant was reduced in virulence. (A) Eight-day-old barley leaves were drop-inoculated with conidium suspensions of the wild type and *chs7* mutant. The concentration of each conidium suspensions was marked to the left. Inoculation with 0.25% gelatin was used as the control. Typical leaves were photographed 5 dpi. (B) Rice sheaths and barley leaves were drop-inoculated with conidia of the wild type and *chs7* mutant. Penetration and invasive hyphae were examined 48 hpi. AP, appressoria; IH, infection hyphae. Bar = 10 µm.

To further confirm that *CHS7* is important for virulence, conidium suspensions with different spore concentrations were drop-inoculated onto barley leaves (Figure 9A). In comparison with the wild type, the *chs7* mutant failed to cause extensive necrosis and chlorosis at 10^4^ conidia/ml or lower. Even at 10^5^ conidia/ml, the *chs7* mutant was less virulent (Figure 9A). In penetration assays with rice leaf sheaths and barley leaves, the wild type produced branching invasive hyphae in plant cells 48 hours post inoculation (hpi) (Figure 9B). Under the same conditions, the *chs7* mutant had only limited invasive hyphae in underlying plant cells (Figure 9B), indicating that *CHS7* is important for invasive growth in *M. oryzae*. Expression of the *CHS7*-*eGFP* suppressed the defects of the observed mutant defects, including appressorium formation and invasive growth.

### *CHS1* is important for infection-related morphogenesis

**Figure 10:**
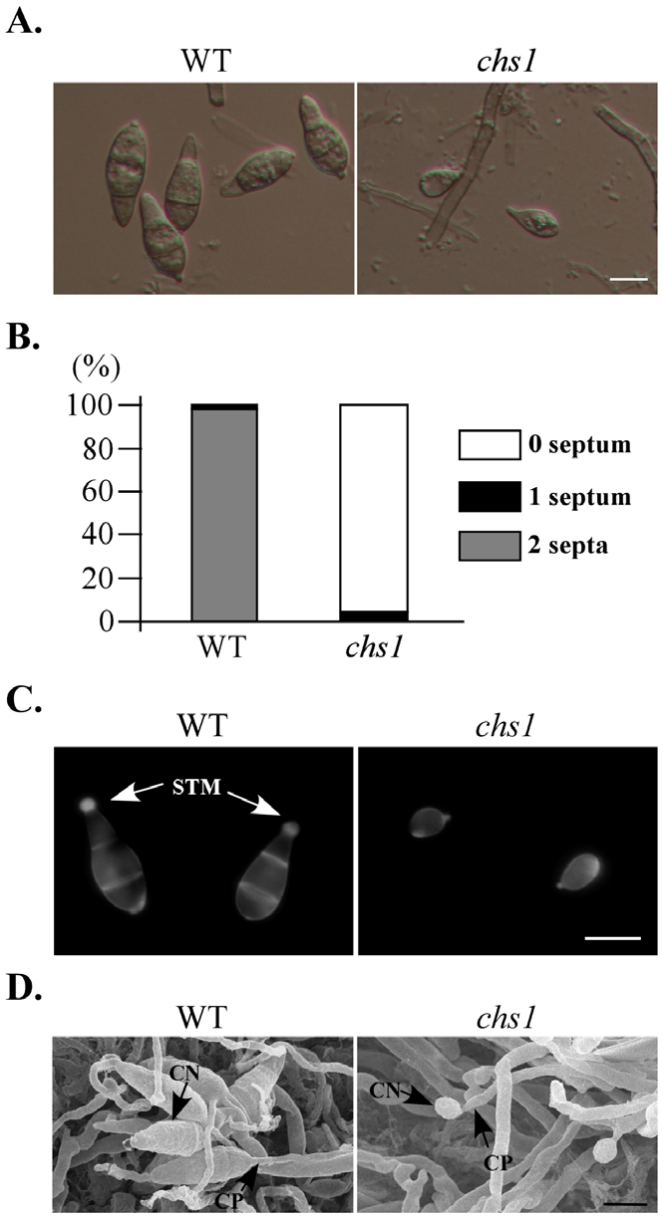
*CHS1* is important for conidiogenesis. (A) Typical conidia of the wild-type strain P131 and *chs1* mutant were examined under differential interference contrast (DIC) microscope. (B) The percentage of conidia with 0-, 1-, and 2-septa in the wild-type strain P131 and *chs1* mutant. The *chs1* mutant failed to produce normal, three-celled pyriform conidia. (C) Spore tip mucilage of the conidia of the wild type and *chs1* mutant were stained with Calcofluor White (CFW) and examined by DIC and epifluorescence (UV) microscopy. Arrows pointed to STM. Bar = 10 µm. (D) Conidia and conidiophores of the wild-type P131 and *chs1* mutant examined under scanning electron microscope (SEM). CO, conidium; CP, conidiophores; STM spore tip mucilage. Bar = 10 µm.

The *chs1* mutant had severe defects in conidium morphology. More than 90% of *chs1* conidia had no septum (Figure 10A; Figure 10B). No three-celled conidium was observed in the *chs1* mutant. The shape of *chs1* conidia also differed from that of the wild type (Figure 10A). One distinct defect of the *chs1* mutant was the lack of spore tip and spore tip mucilage (STM) because the top of conidia were rounded (Figure 10A). When conidia were stained with Calcofluor white (CFW), the STM of the wild-type conidia had strong fluorescence signals. Conidia of the *chs1* mutant lack a clear tip and STM staining (Figure 10C). When examined by scanning electron microscope (SEM), the *chs1* mutant produced conidia sympodially on conidiophores (Figure 10D), indicating that *CHS1* may be dispensable for conidiophore development and the initiation of conidiogenesis. However, later stages of conidiogenesis were blocked or defective in the *chs1* mutant after the initial development of young conidia. *CHS1* may be responsible for the synthesis of the apical landmark in the developing conidia.

**Figure 11:**
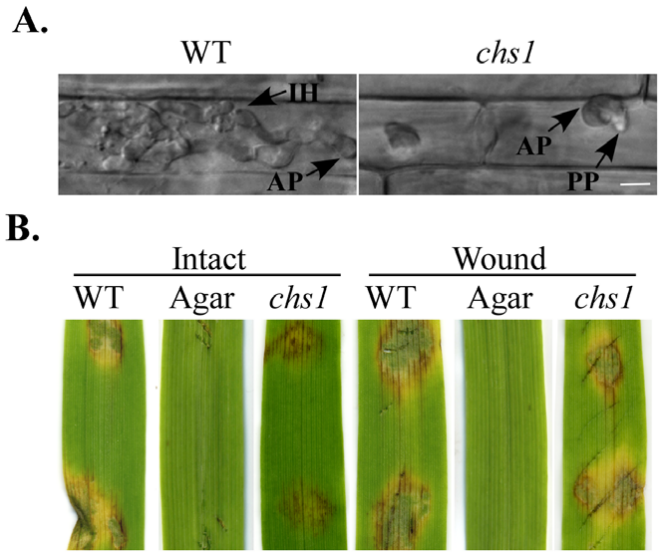
The *chs1* mutant was defective in appressorium penetration and plant infection. (A) Appressorium penetration assays with rice leaf sheaths. Appressoria of the *chs1* mutant failed to penetrate plant cells and develop infectious hyphae at 48 hpi. (B) Intact and wounded barley leaves were inoculated with culture blocks of the wild-type P131 and *chs1* mutant. Lesions were visible 5 dpi after removing the inoculum. Inoculation with water agar blocks was the negative control. AP, appressorium; IH, invasive hyphae; PP, penetration peg.

Although the *chs1* mutant was reduced in conidium germination and appressorium formation, nuclear division and migration appeared to be normal during appressorium formation (Figure S4). Because appressoria formed by *chs1* germ tubes were smaller than those of the wild-type appressoria, we conducted penetration assays with rice leaf sheaths (Figure 11A). As expected, the *chs1* mutant formed melanized appressoria but failed to develop invasive hyphae at 48 h in the epidermal cells of rice leaf sheaths, although penetration pegs may be present. Under the same conditions, the wild type produced branching, bulbous invasive hyphae (Figure 11A).

We then assayed the virulence of the *chs1* mutant on detached barley leaves. On intact leaves, culture blocks of the wild type cause extensive necrosis surrounding the inoculation site but the *chs1* mutant only caused limited necrosis directly beneath culture blocks (Figure 11B). On wounded barley leaves, the wild type and *chs1* mutant caused lesions of similar sizes at the wounding sites (Figure 11B). These results indicate that the reduction of the *chs1* mutant in virulence may be directly related to its defect in appressorium penetration.

### *CHS1* is highly expressed in young, developing conidia

**Figure 12:**
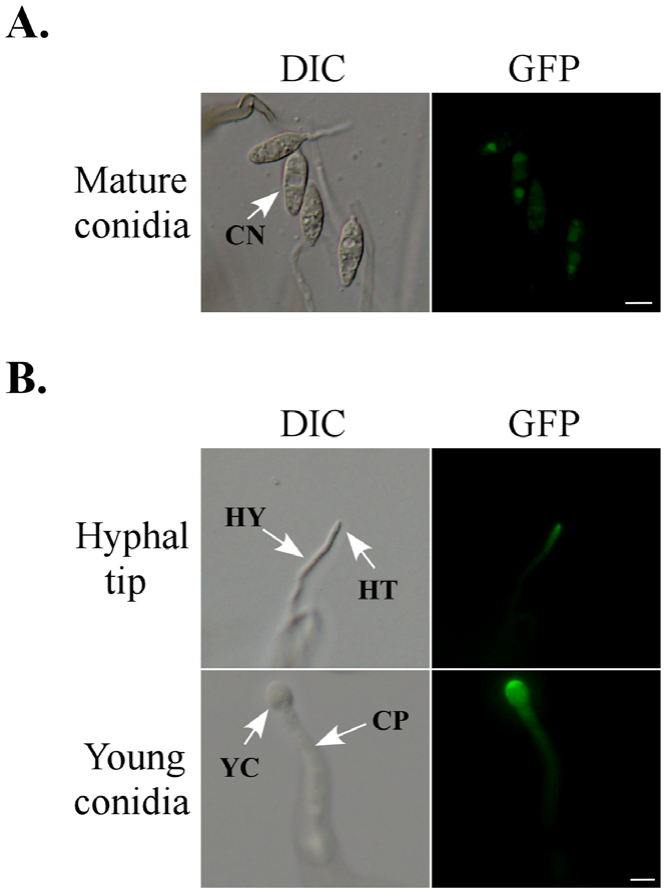
Chs1-eGFP fusion proteins accumulate to the tips. (A) Conidia produced by transformant LA33 expressing the *CHS1-eGFP* construct were observed by DIC and epifluorescence microscopy. (B) Hyphal tips and developing conidia of transformant LA33. The Chs1-eGFP fusion protein localized to the tip region in vegetative hyphae (the upper panel) and developing a young conidium (the bottom panel) at the tip of a conidiophore. CO, conidia; CP, conidiophores; HT, hyphal tips; HY, hyphae; YC, young conidia. Bar = 10 µm.

To determine the expression and localization of *CHS1*, a *CHS1*-e*GFP* fusion construct under the control of its native promoter was generated and transformed into the *chs1* mutant LA40 (Table 1). Transformant LA33 (Table 1) was identified by PCR and confirmed by Southern blot analysis to contain a single copy of pLA8. It had no obvious defects in vegetative growth, appressorium formation, penetration, and plant infection. Moreover, the conidium morphology and conidiation defects of the *chs1* mutant were rescued in transformant LA33 (Figure 12A). In infection assays, transformant LA33 was as virulent as the wild type (Figure 8), indicating that the GFP fusion had no effect on *CHS1* function and the *CHS1*-e*GFP* fusion complemented the *chs1* mutant in virulence.

In transformant LA33 (Table 1), weak GFP signals were detected in the cytoplasm and vacuoles of vegetative hyphae, conidia (Figure 12A), and infectious hyphae (data not shown). Most likely, Chs1-eGFP fusion proteins that normally localize to the inner side of cytoplasm membrane were internalized and possibly degraded in these tissues. GFP signals were much stronger at the tip region of hyphae and conidiophores (Figure 12B). In developing conidia, the distal end region had the strongest GFP signals (Figure 12B).

### Deletion of *CHS6* blocks appressorium penetration and invasive growth

**Figure 13:**
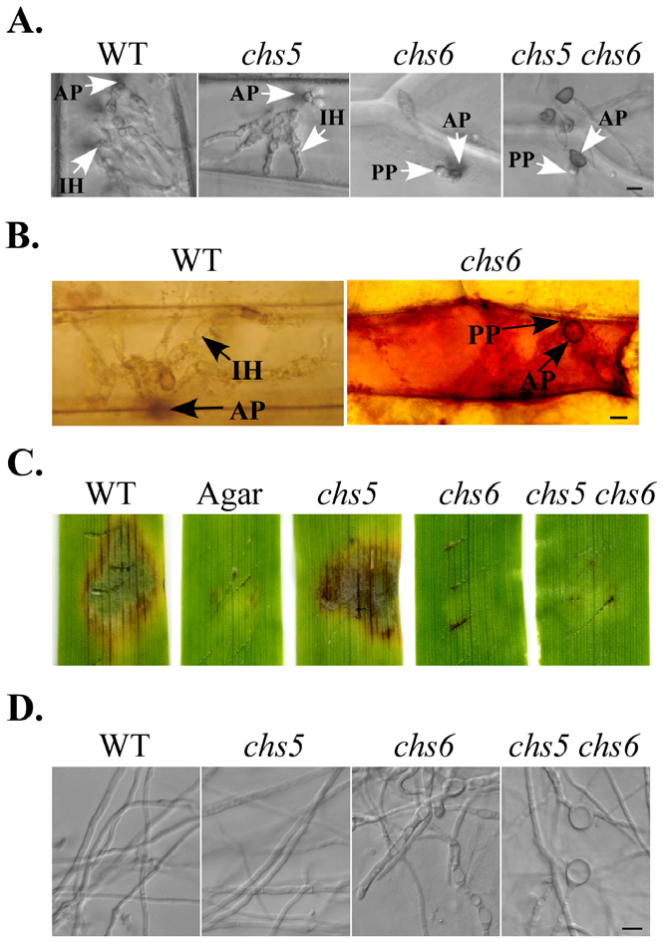
The *chs6* mutant and *chs5 chs6* double mutant failed to penetrate the plant cell. (A) Appressorium penetration assays with onion epidermal cells. Appressoria of the *chs6* mutant and *chs5 chs6* double mutant failed to penetrate plant cells and develop infectious hyphae at 48 hpi. Bar = 10 µm. (B) ROS accumulation in the infected barley leaves by the *chs6* mutant stained with DAB. Strong ROS accumulations were observed in the leaves inoculated with the *chs6* mutant. Bar = 10 µm. (C) Wounded barley leaves were inoculated with culture blocks of the wild-type P131, *chs5*, *chs6*, and *chs5 ch6* double mutant. Lesions were visible 5 dpi after removing the inocula. Inoculation with water agar blocks was the negative control. (D) Vegetative hyphae of the wild-type P131 and the *chs5*, *chs6*, and *chs5 chs6* mutants from 2-day-old 5×YEG cultures. Bar = 10 µm. AP, appressorium; IH, invasive hyphae; PP, penetration peg.

Because the *chs6* mutant still formed appressoria but was non-pathogenic (Figure 3; Figure 8), we then assayed its defects in penetration and invasive growth. Similar to what was observed on artificial surfaces, the *chs6* mutant had no defects in appressorium formation on onion epidermal cells. However, appressoria formed by the mutant failed to successfully penetrate and develop invasive hyphae in plant cells 48 hpi (Figure 13A), which was consistent with spray infection results (Figure 8). Under the same conditions, extensive invasive hyphae were formed by the wild type. When stained with 3,3′-diaminobenzidine (DAB), strong ROS accumulation was observed only in plant cells penetrated by the *chs6* mutant (Figure 13B). The *chs6* mutant appeared to develop penetration pegs and elicited cytoplasm aggregation in underlying plant cells (Figure 13A), indicating that the *chs6* penetration was blocked by plant defense responses [42].

To further characterize the defects of the *chs6* mutant in plant infection, we conducted wound-inoculation assays with barley leaves. On wounding sites inoculated with culture blocks of the wild type, extensive necrosis was observed at the wounding sites and in the surrounding area (Figure 13C). The *chs6* mutant only caused limited darkening at the wounding site. No extensive, spreading zone of necrosis was observed (Figure 13C). Therefore, the *chs6* mutant was defective in the differentiation and growth of invasive hyphae *in planta*. To confirm that these phenotypes were directly related to the deletion of *CHS6*, we conducted co-segregation analysis. Among 42 progeny isolated from the LA26 (*chs6*)×S1528 (WT) genetic cross (Table 1), 22 of them were resistant to hygromycin and had the same defects with the *chs6* mutant. The rest 20 hygromycin-sensitive progeny were similar to the wild type.

### The *chs5 chs6* double mutant has more severe defects than the *chs6* mutant

Because the *CHS5* and *CHS6* genes have similar structures and expression profiles, to further characterize their relationship, we generated the *chs5 chs6* double mutant by deleting the 7.2-kb fragment containing the N-terminal portions of these two chitin synthase genes (Figure S2H). The double mutant LA49 (Table 1) was confirmed by Southern blot analysis (Figure S2H). Although the *chs5* mutant had no obvious defects in growth or conidiation, the *chs*5 *chs*6 double mutant had more severe defects in vegetative growth (Figure 3A; Figure S5; Table S3) and conidiation (Table 2) than the *chs6* mutant. Unlike colonies of the *chs6* mutant with a wrinkled surface, colonies formed by the double mutant were smaller in size and had smooth appearance (Figure 3A). When examined under DIC microscope, the hyphal growth defects observed in the *chs6* mutant also was enhanced in the double mutants (Figure 13D). The intercalary and apical hyphal swellings tended to be larger in the double mutant than the *chs6* mutant (Figure 13D). These results indicate that *CHS5* and *CHS6* have overlapping functions in maintaining polarized growth in vegetative hyphae.

Same as the *chs6* mutant, the *chs5 chs6* double mutant formed melanized appressoria (Figure 3B). No obvious defects were observed in appressorium formation. In spray infection assays with both rice and barley leaves, the *chs5 chs6* double mutant was non-pathogenic (Figure 8). In penetration assays with onion epidermal cells, the *chs5 chs6* mutant, like the *chs6* mutant failed to successfully penetrate underlying plant cells and develop invasive hyphae 48 hpi (Figure 13A). In wound-inoculation assays, the wild type and *chs5* mutant caused extensive necrosis at the wounding sites and surrounding areas. The *chs5 chs6* mutant caused limited darkening at the wounding sites but failed to cause extensive necrosis in the surrounding areas (Figure 13C). The expression levels of *CHS3* and *CHS4* were increased 2.6- and 2.4-fold, respectively, in the *chs5 chs6* double mutant (Figure S6). Increased expression of these *CHS* genes may be able to partially compensate for the deletion of both *CHS5* and *CHS6* genes.

## Discussion

Chitin and glucan are two major components of fungal cell wall. The *F. graminearum* and *A. fumigatus* genomes have eight *CHS* genes [1], [43]. Like *N. crassa*, *M. oryzae* has seven predicted chitin synthase genes. The *CHS1* gene was highly expressed in conidia, especially in young conidia in *M. oryzae* (Figure 12). Deletion of *CHS1* resulted in the production of conidia with a defective morphology. Majority of *chs1* conidia were globular, single-celled (Figure 10). It appears that *CHS1* plays a conidium-specific role in cytokinesis in developing conidia. *CHS1* may be responsible for the synthesis of the landmark for the apical cell during conidiogenesis. In *N. crassa*, the *chs-1* ^RIP^ mutant had no defects in conidiogenesis [18]. In *A. nidulans*, there are two class I chitin synthase genes, *chsB* and *chsF* [3]. Although ChsB functions in conidiophore and conidium development, the *chsB* mutant has normal conidium morphology [23]. In *A. fumigatus*, deletion of *chsC* has no detectable phenotype. The *chsG* and *chsC chsG* mutants were defective in hyphal growth and conidiation but produced normal conidia [44].

As the inocula for dispersal in the field, conidia play a critical in the initiation of plant infection and spreading. In this study, we found that three *CHS* genes, *CHS1*, *CHS2* and *CHS* 6 are important in conidiation of *M. oryzae*. In particular, the *chs1* mutant produced single cell conidia. Because conidia produced by *N. crassa* and *Aspergilli* are single-celled, defects of the *chs1* mutant may be unique to cell division and cytokinesis in developing conidia. To test this hypothesis, we generated the *Fgchs1* (FGSG_10116.3) deletion mutant in *F. graminearum*. Our preliminary data indicate that the *Fgchs1* mutant were shorter and narrower than those of the wild type. While approximately 80% of the wild-type conidia had 3-4 septa, the majority of *Fgchs1* conidia were three-celled (two septa), indicating that the *CHS1* ortholog also is important for septation in conidia in *F. graminearum*. We would like to point out here that *F. graminearum* has two class I chitin synthase genes (Figure S1). Although FGSG_10116.3 shares higher similarity with the *M. oryzae CHS1* gene than FGSG_03418.3, they may have overlapping functions in *F. graminearum* during conidiogenesis.

For many plant pathogenic fungi, conidium attachment and germination is the initial infection step. We noticed that many of the single-celled conidia were defective in germination in the *chs1* mutant. Although the wild-type and *chs1* mutant strains had similar chitin content in vegetative hyphae, the latter had higher chitin content in conidia (Figure 4). In consistent with this observation, conidia of the *chs1* mutant had patches of strong CFW staining cell wall areas (Figure 10C). Higher chitin content and thicker cell wall may be responsible for reduced conidium germination in the *chs1* mutant. Deletion of a chitin synthase gene resulted in an increased in overall chitin content has been reported in other fungi, such as the *chs2* mutant of *C. albicans* [45] and the *chsA chsC* double mutant of *A. nidulans* [46].

Among the seven *CHS* genes in *M. oryzae*, *CHS2* had the lowest expression level in conidia, appressoria, and infected leaves. Its expression level in vegetative hyphae was only slightly higher than that of *CHS7* (Figure 2). In *F. oxysporum*, the *chs2* mutant had a significant reduction in virulence [37]. However, the *chs2* mutant of *M. oryzae* was as virulent as the wild type on the seedlings of rice and barley although it was reduced in vegetative growth and conidiation, indicating that *CHS2* was not important for plant infection. Therefore, *CHS2* must have distinct functions in invasive hyphae and vegetative hyphae. In *U. maydis*, there are three class II chitin synthase genes, *CHS2*, *CHS3*, and *CHS4*. The *chs2* mutant has no obvious defects in mating, dimorphism, and plant infection, but deletion of either *chs3* or *chs4* resulted in a significant reduction in mating efficiency and virulence [5], [29]. In *M. oryzae*, the *chs2* mutant had increased sensitivity to Calcofluor white, Congo red, and Nikkomycin Z (Table 3), indicating that *CHS2* is important for cell wall integrity, which was similar to the *Bcchs1* (II) mutant in *B. cinerea* [32].

The *CHS3* and *CHS1* genes had similar structures (Figure 1A) and similar expression patterns (Figure 2B) in *M. oryzae*. In the *chs3* mutant, the *CHS1* expression was slightly up-regulated in the *chs3* mutant (Figure 5). Our preliminary data showed that the *chs1 chs3* double mutant had increased sensitivity to hyperosmotic and oxidative stresses, indicating that the *CHS1* and *CHS3* genes may have compensatory functions in cell wall modifications in responses to stresses in *M. oryzae*.

In *M. oryzae*, deletion of the *CHS3*, *CHS4*, and *CHS5* genes that encode classes III, IV, and V chitin synthases, respectively, had no significant changes in conidiation, appressorium formation, and plant infection although the growth rate might be slightly reduced. In *A. nidulans*, deletion of the *chsC* gene had no obvious effects on growth and reproduction but the *chsA* (II) *chsC* (III) double mutant was defective in septum formation and asexual or sexual development [47]. It is possible that *CHS3* has overlapping functions with *CHS2* or other *CHS* genes in *M. oryzae*. In *Colletotrichum graminicola* and *F. oxysporum*, the class III chitin synthase genes are dispensable for hyphal growth, conidiation, and plant infection [36], [37]. Deletion of *CHS3* and *CHS4* led to up-regulated expression of other *CHS* genes (Figure 5), suggesting that these two genes might function as negative regulators on expression of other *CHS* genes.

Both the *CHS5* and *CHS6* MMD-containing chitin synthase genes had the highest expression level in vegetative hyphae. Like their orthologs in several other filamentous ascomycetes, *CHS5* and *CHS6* are located next to each other in a head-to-head arrangement in *M. oryzae* (Figure 1B). Because their ORFs are only 2,957-bp apart, it is possible that *CHS5* and *CHS6* share some regulatory elements in their promoters. Putative Rlm1-binding sites are present in the promoter regions of the *CHS5* and *CHS6* orthologs in *A. nidulans* [6], and *F. oxysporum* [42]. In *S. cerevisiae*, transcription factor Rlm1 functions downstream of the PKC pathway for regulating cell wall integrity [48]. In *M. oryzae*, the *MIG1* gene, an ortholog of *RLM1*, is required for the development and growth of invasive hyphae in plant cells [49]. The *chs6* mutant had similar defects in plant infection (Figure 8) with the *mig1* mutant [49]. It is possible that the expression of *CHS6* may be under the control of Mig1 in *M. oryzae*.

In *A. nidulans*, the *csmA* and *csmB* single deletion mutants had no significant changes in the growth rate but the *csmA csmB* double mutant was not viable [6], suggesting an essential role for these *CHS* genes in hyphal tip growth [50], [51]. In *F. oxysporum*, the *chsV chsVb* double mutant is viable but both *chsV* and *chsVb* single mutants were non-pathogenic [39]. Similar results were reported in *F. graminearum*. Both *GzCHS5* and *GzCHS7* are important for hyphal growth and pathogenicity [34]. In *M. oryzae*, deletion of *CHS5* had no effects on vegetative growth and plant infection but the *chs6* mutant was non-pathogenic and defective in hyphal growth. The *chs5 chs6* double mutant was more significantly reduced in vegetative growth and conidiation than the *chs6* mutant, indicating that *CHS5* may play a minor overlapping role with *CHS6* in growth and differentiation. Hyphal swelling observed in the *chs6* and *chs5 chs6* mutants indicated a weakened cell wall. Chs5 is 92-amino-acid residues shorter than Chs6 in the MMD domain. It lacks the consensus motifs of myosins, such as the P-loop, Switch I, and Switch II, that are present in Chs6 (Figure 1B), suggesting that functional differences of these two genes may derive from their structural differences in their MMDs.

Although there was a previous report that appressorium formation by germ tubes was blocked in the *chs7* mutant [41], we found that its hyphal tips had no defects in developing melanized appressoria on artificial hydrophobic surfaces, suggesting that the role of *CHS7* in appressorium formation is cell type-specific. It is possible that germ tubes, but not hyphal tips, of the *chs7* mutant are defective in surface recognition. Recently, it has been shown that the *msb2* mutant was defective in appressorium formation on artificial surfaces but formed appressoria efficiently on plant surfaces [52]. We also found that germ tubes of the *chs7* mutant formed melanized appressoria on plant surfaces (Figure 6). These results indicate that the *CHS7* gene is not essential for appressorium formation and defects of the *chs7* mutant in appressorium formation are surface-dependent and cell-type specific. Appressorium formation by the hyphal tips and germ tubes may require different chitin synthase genes in *M. oryzae*. Therefore, it will be important to further characterize the role of *CHS7* in appressorium formation by germ tubes and hyphal tips. It is also critical to identify and characterize the *CHS* gene(s) with elevated expression levels in the germ tubes or hyphal tips of the *chs7* mutant during appressorium formation on different surfaces.

To our knowledge, systemic characterization of chitin synthase genes in fungal pathogens has been carried out only in *U. maydis*, a basidiomycetous fungal pathogen. Our functional characterization of all the seven predicted chitin synthase genes in *M. oryzae* seems to be the first such study in an ascomycetous phytopathogenic fungus. Although this approach is not novel, we did observe unique phenotypes in the *chs1*, *chs6*, and *chs7* mutants that are not reported in previous studies. *CHS1* is the first fungal chitin synthase gene that specifically affects conidium morphology and germination. The fact that the *chs1* mutant produced more conidia than the *chs2* and *chs6* mutants suggests that *CHS1* may be dispensable for conidiophore development and play a unique role after the de-limitation of young conidia. Because the chitin content was increased in *chs1* conidia, other *CHS* genes must be able to compensate the deletion of *CHS1* in *M. oryzae*. The *chs5 chs6* double mutant is viable, which also indicate that other *CHS* genes can support hyphal tip growth. Therefore, it will be important to further characterize the interactions and relationships among the *CHS* genes during different developmental and plant infection stages in *M. oryzae*. In conclusion, the chitin synthases important for hyphal growth, conidiogenesis, appressorium development, and pathogenesis could be provided as potential targets to develop new strategies or fungicides to control the rice blast disease.

## Materials and Methods

### Strains and culture conditions

The *M. oryzae* wild-type stains P131 and S1528, and various transformants generated (Table 1) in this study were cultured on oatmeal agar (OTA) at 25°C under bright light [53]–[55]. Fungal genomic DNA isolation, protoplast preparation, transformation, measurements of vegetative growth rate and conidiation were performed as described [52], [56], [57]. For testing sensitivities to various stresses, fungal growth was assayed after incubation at 25°C for 5 days on complete medium (CM) plates with 0.7 M NaCl, 1.2 M sorbitol (Amresco), 50 µg/ml Congo red (Sigma), 50 µg/ml Calcofluor white (Sigma), 5 mM Nikkomycin Z (Sigma) as described [5], [52], [58], [59]. 10 µM cutin monomer 1,16-hexadecanediol (Sigma) was used to examine the effect on appressoria formation by germ tubes or hyphal tips [52], [60]. Examination of GFP expression in young conidia and conidiophores were performed as described [61]. Genetic crosses were performed as described [53], [62], [63].

### Generation of the *CHS* gene replacement constructs and mutants

For generating the *CHS1* gene replacement construct pLA1, the 1.3-kb upstream and 1.7-kb downstream fragments of the *CHS1* gene were amplified with primers 1P1/1P2 and 1P3/1P4 (Table S1), respectively. The resulting PCR products were cloned into the *Spe*I-*Eco*RI and *Sal*I-*Kpn*I sites of pKOV21 [53]. After linearization with *Not*I, pLA1 was transformed into protoplasts of the wild-type strain P131. Putative *chs1* mutants were identified by PCR and further confirmed by Southern blot hybridizations [64]. Similar strategies were used to generate the gene replacement constructs for the other chitin synthase genes.

### qRT-PCR analysis

Total RNAs were isolated from mycelia harvested from 2-day-old minimal media or complete media, conidia harvested from 10-day-old oatmeal agar cultures, 24 h appressoria, and infected rice leave harvested 5 dpi with the TRIzol reagent (Invitrogen) and purified with the DNA-free kit (Ambion). First strand cDNA was synthesized with the AccuScript High Fidelity RT-PCR System (Stratagene) and used as the template for qRT-PCR as described [65], [66]. The *M. oryzae* actin gene MGG_03982.6 was used as the endogenous control for normalization. Relative expression levels were estimated with the 2^−*ΔΔ*Ct^ method [67]. Data from three biological replicates were used to calculate the mean and standard deviation. Primers used for qRT-PCR are listed in Table S1.

### Measurement of the chitin content

The chitin content was determined by measuring the amount of glucosamine released by acid hydrolysis of fungal cell wall [68]–[70]. One gram of freshly harvested vegetative hyphae or conidia was grinded in liquid nitrogen and suspended in 5 ml of deionized water. After centrifugation at 13,000 g for 10 min at 4°C, the pellets were freeze-dried overnight (Labconco). For each 5 mg of the dried pellet, 1 ml of 6 M HCl was added. After hydrolyzed at 100°C for 4 h, the hydrolysate was adjusted with 10 N NaOH to pH 7.0. An aliquot (0.2 ml) of the resulting mixture was added to 0.25 ml of 4% acetylacetone in 1.25 M sodium carbonate and heated for 30 min at 100°C. The mixture was heated for 1 h at 60°C after adding 2 ml of ethanol and 0.25 ml of the Ehrlich reagent [71] and centrifugated at 13,000 g for 10 min. The supernatant was measured for absorbance at 530 nm (BioTek). The chitin content (µg of glucosamine/mg of dry weight of fungal biomass) was calculated in accordance with a standard curve established by measuring the absorbance of known amounts of glucosamine hydrochloride (Sigma).

### Chitin synthase activity assay

Microsomal fractions of proteins were isolated with a similar procedure as described [32], [70], [72] with minor modifications. Homogenates of 1 g of mycelia grinded in liquid nitrogen were suspended in 20 ml of 50 mM Tris-HCl (pH 7.4) and 2.5 mM MgCl_2_. After centrifugation at 13,000 g for 10 min, the supernatant was centrifugated at 40,000 g for 1 h at 4°C. The resulting pellet was dissolved in 0.5 ml of 50 mM Tris-HCl (pH 7.4) with 30% glycerol (w/v) and assayed for protein contents with a NanoDrop (Thermo). Chitin synthase activities were assayed as described [18], [72] in a 50 µl of reaction mixture containing 10 mM Tris-HCl (pH 7.4), 10 mM MgCl_2_, 1.0 mM UDP-N-acetyl-D-glucosamine, 667 nM (0.01 uCi) UDP-N-acetyl-D-[U-^14^C]glucosamine (300 mCi/mmol; American Radiolabled Chemicals), and 32 mM N-acetyl-glucosamine. After incubation at 30°C for 1 h, the reactions were terminated by adding 50 µl of acetic acid. The final mixture was loaded onto a glass fiber filter (Φ 2.5 cm, GF/C, Whatman) and washed three times with 1 ml of 30% acetic acid and 70% ethanol, and once with 1 ml of ethanol. The discs were dried and the radioactivity retained on the filters was measured by a liquid scintillation counter (PerkinElme). The chitin synthase activity was expressed as nM of GlcNAc incorporated into chitin per mg of protein per minute [32].

### Generation of the *CHS1-eGFP* construct and transformant

A 4.5-kb fragment of *CHS1* gene along with its 1.5-kb promoter region was amplified with primers DW1F/DW1R (Table S1) and cloned into pKNTG [53]. The resulting *CHS1*-*eGFP* fusion construct pLA8 was confirmed by sequencing analysis. After *Not*I digestion, pLA8 was transformed into protoplasts of the *chs1* mutant LA40 (Table 1). Monoconidial cultures of neomycin-resistant transformants were screened for GFP expression and confirmed by Southern blot analysis. Similar strategies were used to generate the *CHS7-eGFP* construct and transformant.

### Assay for appressorium formation and plant infection

Appressorium formation by germ tubes on artificial surface were assayed as described [52], [54], [73]. Appressorium formation assays with hyphal tips were performed as described [74]. Appressorial penetration and invasive hyphae development were assayed with rice leaf sheaths and onion epidermal cells [53], [64], [75]. For appressoria formation and penetration assay on plant surface, 20 µl of conidia suspension or fresh hyphal bocks were dropped on the 5-day-old barley leaf cultivar, Golden Promise, and incubated within a moist chamber for 24 h and 48 h, respectively. In order to observe the appressoria and invasive hyphae clearly, 96% ethanol (v/v) were used to remove the chlorophyll [76]. After shaking the sample in 96% ethanol (v/v) for 12 h, a bright microscope was used to observe the appressoria and invasive hyphae. For testing the appressoria formed by hyphal tips on rice root surface, 5-day-old root of rice cultivar, LTH was placed on the surface of MS (Calsson Lab) media with 1% agar [77], [78], and the fresh hyphal blocks were placed on the root surface. Appressoria formation was examined with a bright microscope at 48 hpi.

For spray infection assays, conidia were adjusted to 1×10^5^ conidia/ml in 0.25% gelatin and used for inoculation with 14-day-old seedlings of rice cultivar, LTH and 8-day-old seedlings of barley cultivar, Golden Promise [53], [79]. Lesion formation was examined at 5–7 days post-inoculation. Mean number of lesions formed on 5-cm leaf tips was determined as described [63], [80]. Infection assays with mycelial blocks on intact or wounded barley leaves were preformed as described [54]. Infection assay with dilution-drop conidia suspension was performed as previously described [81].

### Scanning electron microscope (SEM) examination

Culture blocks from 10-day-old OTA cultures were fixed in 4% glutaraldehyde at 4°C for 16 h. The samples were then dehydrated and coated with gold as described [52], [82]. Conidia and conidiophores were observed with a JSM-6360LV (Jeol Ltd., Tokyo) scanning electron microscope.

### Calcofluor white, DAPI staining, and DAB assays

Conidia and hyphae were fixed in a fixation solution (3.75% formaldehryde, 50 mM PBS, pH 7.5, 0.2% Triton X-100) for 15 min and then stained with 10 µg/ml Calcoflour white and/or 1 µg/ml DAPI (Sigma) for 5 min [54], [83]. After washing twice with sterile distilled water, the chitin deposition in cell wall and septa, and neclear devision were examined with an Eclipse 800 epifluorescence microscope (Nikon). For staining with 3,3′-diaminobenzidine (DAB, Sigma), 5-day-old barley leaves were inoculated with the wild type and *chs* mutant. After incubation at room temperature for 48 h, samples were stained with 1 mg/ml DAB solution (pH 3.8) for 8 h and de-stained with ethanol/acetic acid (94/4, v/v) for 1 h before examination [65], [76], [84].

### Accession numbers

Sequence data from this article can be found in the GenBank/EMBL data libraries under accession number JF912404 (*CHS1*), JF912405 (*CHS2*), JF912406 (*CHS3*), JF912407 (*CHS4*), JF912408 (*CHS5*), JF912409 (*CHS6*), and EU935590 (*CHS7*).

## Supporting Information

Figure S1 **Phylogenetic tree of fungal chitin synthase.** Phylogenetic tree was generated by the Clustalx1.83. The scale bar indicates 0.1 distance units. Species abbreviation are *Af* (*Aspergillus fumigatus)*, *An* (*Aspergillus nidulans*), *Bc* (*Botrytis cinerea*), *Cg* (*Colletotrichum graminicola*), *Fo* (*Fusarium oxysporum*), *Gz* (*Gibberella zeae*), and *Nc* (*Neurospora crassa*).
(TIF)

Figure S2 ***CHS*** **gene deletion construct and confirmation.** (A) *CHS1* gene deletion strategy and confirmation. *CHS1* deletion construct (the upper) was created by replacing the *CHS1* gene with hygromycin phosphotransferase cassette (*hph*). The upstream and downstream flanking sequences were amplified with primers 1P1/1P2 and 1P3/1P4, and ligated with the *hph* cassette. The positions of primers 1P1, 1P2, 1P3, 1P4, 1out, and PF are indicated with small arrows. E, *Eco*RI; EV, *Eco*RV; K, *Kpn*I; S, *Sal*I; Sp, *Spe*I. This construct was introduced into the wild-type strain P131. The putative mutants were screened with primers 1out/PF, and the putative mutants LA11 and LA40 had 1.9-kb specific band. 0.2-kb fragment within *MoACT1* gene was amplified as the endogenous reference (left bottom). Southern blot of *Eco*RV-digested genomic DNA of wild-type P131 and *chs1* mutant LA11 and LA40 hybridized with Probe 1, which was amplified with primers 1P1/1P2. The results show a single 5.0-kb band (lane1) for the wild-type P131 and a single 6.0-kb band (lane 2 and lane 3) for *chs1* mutant LA11 and LA40 (right bottom). (B) *CHS2* gene deletion strategy and confirmation. *CHS2* deletion construct (the upper) was created by replacing the *CHS2* gene with *hph*. The upstream and downstream flanking sequences were amplified with primers 2P1/2P2 and 2P3/2P4, and ligated with the *hph* cassette. The positions of primers 2P1, 2P2, 2P3, 2P4, 2out, and PF are indicated with small arrows. B, *Bam*HI; E, *Eco*RI; H, *Hin*dIII; K, *Kpn*I. This construct was introduced into the wild-type S1528. The deletion mutants were screened with primers 2out/PF, and the putative mutants LA2 and LA6 had 2.3-kb specific band. 0.2-kb fragment within *MoACT1* gene was amplified as the endogenous reference (left bottom). Southern blot of *Kpn*I-digested genomic DNA of wild-type strain S1528 and *chs2* mutant LA2 and LA6 hybridized with Probe 2, which was amplified with primers 2P1/1P2. The results show a single 3.0-kb band (lane 1) for the wild-type S1528 and a single 6.1-kb band (lane 2 and lane 3) for *chs2* mutant LA2 and LA6 (right bottom). (C) *CHS3* gene deletion strategy and confirmation. *CHS3* deletion construct (the upper) was created by introducing the *hph* within the *CHS3* gene. The fragment within *CHS3* gene was amplified with primers 3P1/3P2. The *hph* gene fragment was amplified with primers 3P3/3P4. The *hph* gene fragment was inserted into the fragment within *CHS3* by *Sal*I restriction enzyme. The positions of primers 3P1, 3P2, 3P3, 3P4, 3out, and PF are indicated with small arrows. B, *Bam*HI; E, *Eco*RI; H, *Hin*dIII; S, *Sal*I; X, *Xho*I. This construct was introduced into the wild-type P131. The deletion mutants were screened with primers 3out/PF, and the putative mutant LA1 had 2.5-kb specific band. 0.2-kb fragment within *MoACT1* gene was amplified as the endogenous reference (left bottom). Southern blot of *Eco*RI and *Hin*dIII-digested genomic DNA of the wild-type P131 and *chs3* mutant (LA1) hybridized with Probe 3, which was amplified with primers 3P3/3P4. The results show a single 6.1-kb band (lane 1) for the *chs3* mutant LA1 and no band (lane 2) for wild-type strain P131 when using *Eco*RI-digesting the genomic DNA; a single 4.7-kb band (lane 3) for the *chs3* mutant LA1 and no band (lane 4) for the wild-type P131 when using *Hin*dIII-digesting the genomic DNA (right bottom). (D) *CHS4* gene deletion strategy and confirmation. *CHS4* deletion construct (the upper) was created by replacing the *CHS4* gene with *hph*. The upstream and downstream flanking sequences were amplified with primers4P1/4P2 and 4P3/4P4, and ligated with the *hph* cassette. The positions of primers 4P1, 4P2, 4P3, 4P4, 4out, and PF are indicated with small arrows. A, *Apa*I; E, *Eco*RI; S, *Sal*I; Sp, *Spe*I. This construct was introduced into the wild-type P131. The deletion mutants were screened with primers 4out/PF, the putative mutants LA3 and LA28 had 2.1-kb specific band. 0.2-kb fragment within *MoACT1* gene was amplified as the endogenous reference (left bottom). Southern blot of *Sal*I-digested genomic DNA of the wild-type P131 and *chs4* mutants LA3 and LA28 hybridized with Probe 4, which was amplified with primers 4P1/4P2. The results show a single 3.5-kb band (lane 1) for the wild-type P131 and a single 4.4-kb band (lane 2 and lane 3) for *chs4* mutants LA3 and LA28 (right bottom). (E) *CHS5* gene deletion strategy and confirmation. *CHS5* deletion construct (the upper) was created by replacing the *CHS5* gene with *hph*. The upstream and downstream flanking sequences were amplified with primers 5P1/5P2 and 5P3/5P4, and ligated with the *hph* cassette. The positions of primers 5P1, 5P2, 5P3, 5P4, 5out, and PF are indicated with small arrows. B, *Bam*HI; Bg, *Bgl*II; E, *Eco*RI; H, *Hin*dIII; K, *Kpn*I; S, *Sal*I. This construct was introduced into the wild-type P131. The deletion mutant was screened with primers 5out/PF, and the putative mutant (LA8) had 2.0-kb specific band. 0.2-kb fragment within *MoACT1* gene was amplified as the endogenous reference (left bottom). Southern blot of *Bgl*II and *Hin*dIII-digested genomic DNA of wild-type P131 and *CHS5* deletion mutant LA8 hybridized with Probe 5, which was amplified with primers 5P3/5P4. The results show a single 6.2-kb band (lane 1) for the *CHS5* deletion mutant LA8 and no band (lane 2) for the wild-type P131 when using *Bgl*II-digesting the genomic DNA; a single 2.2-kb band (lane 3) for the *chs5* mutant (LA8) and no band (lane4) for the wild-type P131 when using *Hin*dIII-digesting the genomic DNA (right bottom). (F) *CHS6* gene deletion strategy and confirmation. *CHS6* deletion construct (the upper) was created by replacing the fragment within *CHS6* gene with *hph*. The upstream and downstream flanking sequences were amplified with primers 6P1/6P2 and 6P3/6P4, and ligated with the *hph* cassette. The positions of primers 6P1, 6P2, 6P3, 6P4, 6out, and PF are indicated with small arrows. E, *Eco*RI; H, *Hin*dIII; K, *Kpn*I; S, *Spe*I. This construct was introduced into the wild-type P131. The deletion mutant was screened with primers 6out/PF, and the putative mutants LA14 and LA26 had 2.0-kb specific band. 0.2-kb fragment within *MoACT1* gene was amplified as the endogenous reference (left bottom). Southern blot of *Eco*RI-digested genomic DNA of wild-type strain P131 and *chs6* mutants LA14 and LA26 hybridized with Probe 6, which was amplified with primers 6P1/6P2. The results show a single 3.5-kb band (lane 3) for the wild-type P131 anda single 4.4-kb band (lane 1 and lane 2) for *chs6* mutants LA14 and LA26 (right bottom). (G) *CHS7* gene deletion strategy and confirmation. *CHS7* deletion construct (the upper) was created by replacing fragment within *CHS7* gene with *hph*. The upstream and downstream flanking sequences were amplified with primers 7P1/7P2 and 7P3/7P4, and ligated with the *hph* cassette. The positions of primers 7P1, 7P2, 7P3, 7P4, 7out, and PR are indicated with small arrows. E, *Eco*RI; H, *Hin*dIII; K, *Kpn*I; P, *Pst*I; S, *Spe*I. This construct was introduced into the wild-type P131. The deletion mutant was screened with primers 7out/PR, and the putative mutant LA12 had 1.7-kb specific band. 0.2-kb fragment within *MoACT1* gene was amplified as the endogenous reference (left bottom). Southern blot of *Eco*RI and *Pst*I-digested genomic DNA of the wild-type P131 and *chs7* mutant LA12 hybridized with Probe 7, which was amplified with primers 7P3/7P4. The results show a single 6.2-kb band (lane 1) for the *chs7* mutant LA12 and no band (lane 2) for wild-type P131 when using *Bgl*II-digesting the genomic DNA; a single 2.9-kb band (lane 3) for the *chs7* mutant LA12 and no band (lane 4) for the wild-type P131 when using *Pst*I-digesting the genomic DNA (right bottom). (H) *CHS5 CHS6* gene deletion strategy and confirmation. The *CHS5 CHS6* deletion construct was generated by replacing the N-terminal regions of both *CHS5* and *CHS6* with the *hph* cassette. The upstream and downstream flanking sequences were amplified with primers 8P1/8P2 and 8P3/8P4, and ligated with the *hph* cassette. B, *Bam*HI; E, *Eco*RI; H, *Hin*dIII; K, *Kpn*I. The putative double mutants LA49 and LA66 were identified by PCR with primers 8out/PF with a 1.8-kb specific band. A 0.2-kb band within *MoACT1* gene was amplified as the endogenous reference (left bottom). Southern blot of *Pst*I-digested genomic DNA of the wild-type P131 and *chs5 chs6* double mutants LA49 and LA66 hybridized with Probe 8, which was amplified with primers 8P3/8P4. The results show a single 2.7-kb band for the wild-type P131 and a single 4.2-kb band for *chs5 chs6* double mutants LA49 and LA66 (right bottom).
(TIF)

Figure S3 **Hydrophobicity assay.** Drops of 20 µl sterile distilled water were placed on the surface of vegetative colony from 7-day-old complement media agar cultures. The results show the surface of vegetative colony from the wild-type P131 and S1528, and seven *CHS* gene deletion mutants were hydrophobic.
(TIF)

Figure S4 **Nuclear division and movement during appressorium formation in the** ***chs1*** **mutant.** Conidia of the wild type and *chs1* mutant incubated on hydrophobic glass coverslips for 0, 1, 4, and 24 h were stained with CFW and DAPI and examined with an epifluorescence microscope. Bar = 10 mm.
(TIF)

Figure S5 **The** ***chs5 chs6*** **double mutant was more significantly reduced in growth rate than** ***chs6*** **mutant.** The wild-type P131, *chs5*, *chs6*, and *chs5 chs6* double mutant were cultured on OTA plates under light at 25°C. Photographs were taken at 7 and 14 dpi, respectively. Bar = 10 mm.
(TIF)

Figure S6 **qRT-assays with the expression levels of** ***CHS*** **genes in the wild-type P131 and** ***chs5 chs6*** **mutants.** The RNA was isolated from vegetative mycelia shaken in CM for two days. The actin gene was used as the endogenous control for normalization. Relative expression levels were estimated with the 2^−ΔΔCt^ method. The expression level of each *CHS* gene in the wild type was arbitrarily set to 1. Mean and standard errors were determined with data from three independent replicates.
(TIF)

Table S1 **PCR primers used in this study.**
(DOC)

Table S2 **The effects of cutin monomer treatment on appressorium formation by the wild type and** ***chs7*** **mutant on different surfaces.**
(DOC)

Table S3 **Defect of the** ***chs*** **5** ***chs*** **6 mutants in vegetative growth.**
(DOC)
