# Supplementary figures and images for: Different Chitin Synthase Genes Are Required for Various Developmental and Plant Infection Processes in the Rice Blast Fungus Magnaporthe oryzae
Source: PLoS Pathog. 2012 Feb 9;8(2):e1002526. doi: 10.1371/journal.ppat.1002526 (PMC3276572; doi:10.1371/journal.ppat.1002526)

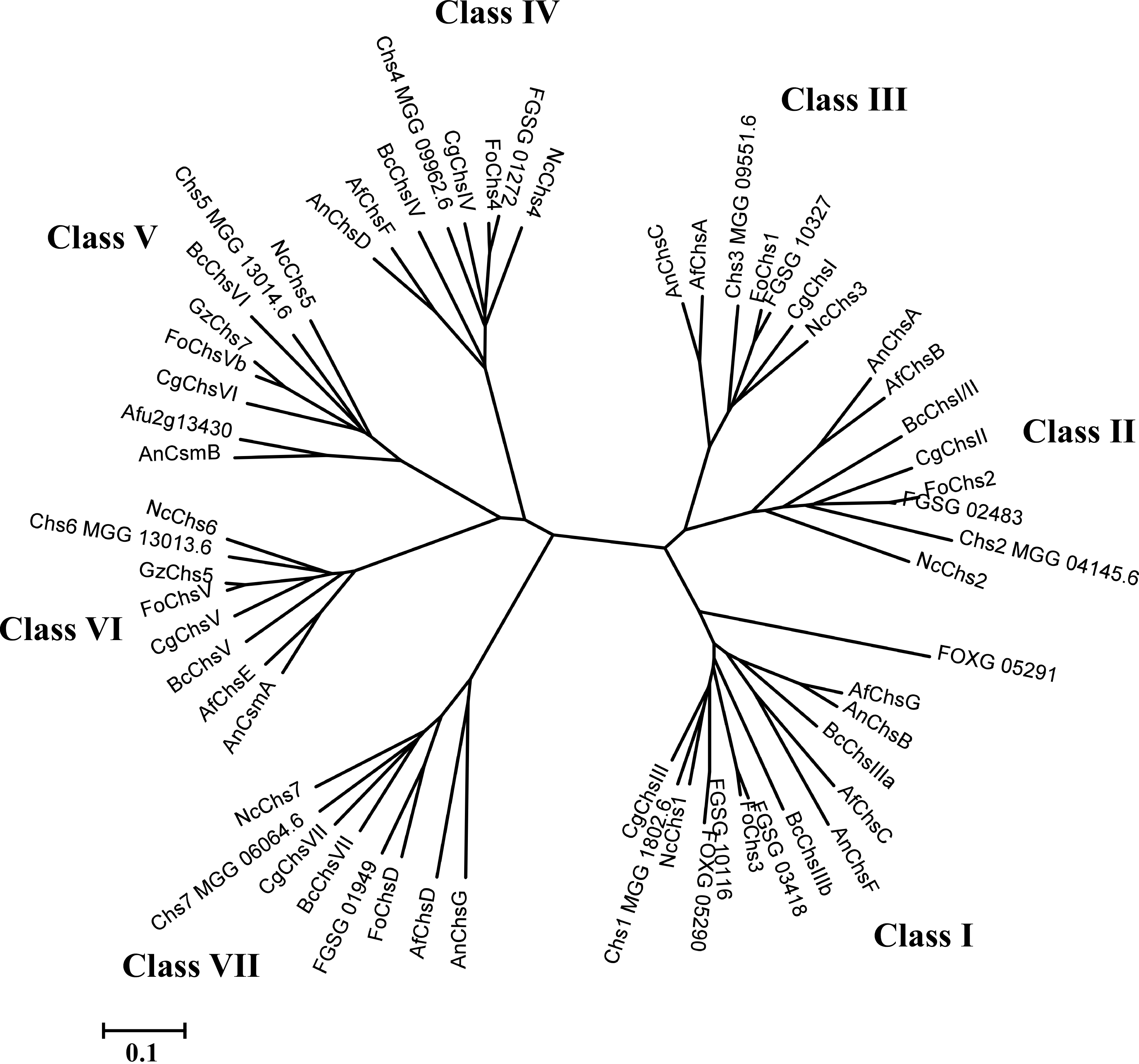

Supplement: Figure S1 — Phylogenetic tree of fungal chitin synthase. Phylogenetic tree was generated by the Clustalx1.83. The scale bar indicates 0.1 distance units. Species abbreviation are Af (Aspergillus fumigatus), An (Aspergillus nidulans), Bc (Botrytis cinerea), Cg (Colletotrichum graminicola), Fo (Fusarium oxysporum), Gz (Gibberella zeae), and Nc (Neurospora crassa). (TIF) [file ppat.1002526.s001.tif]

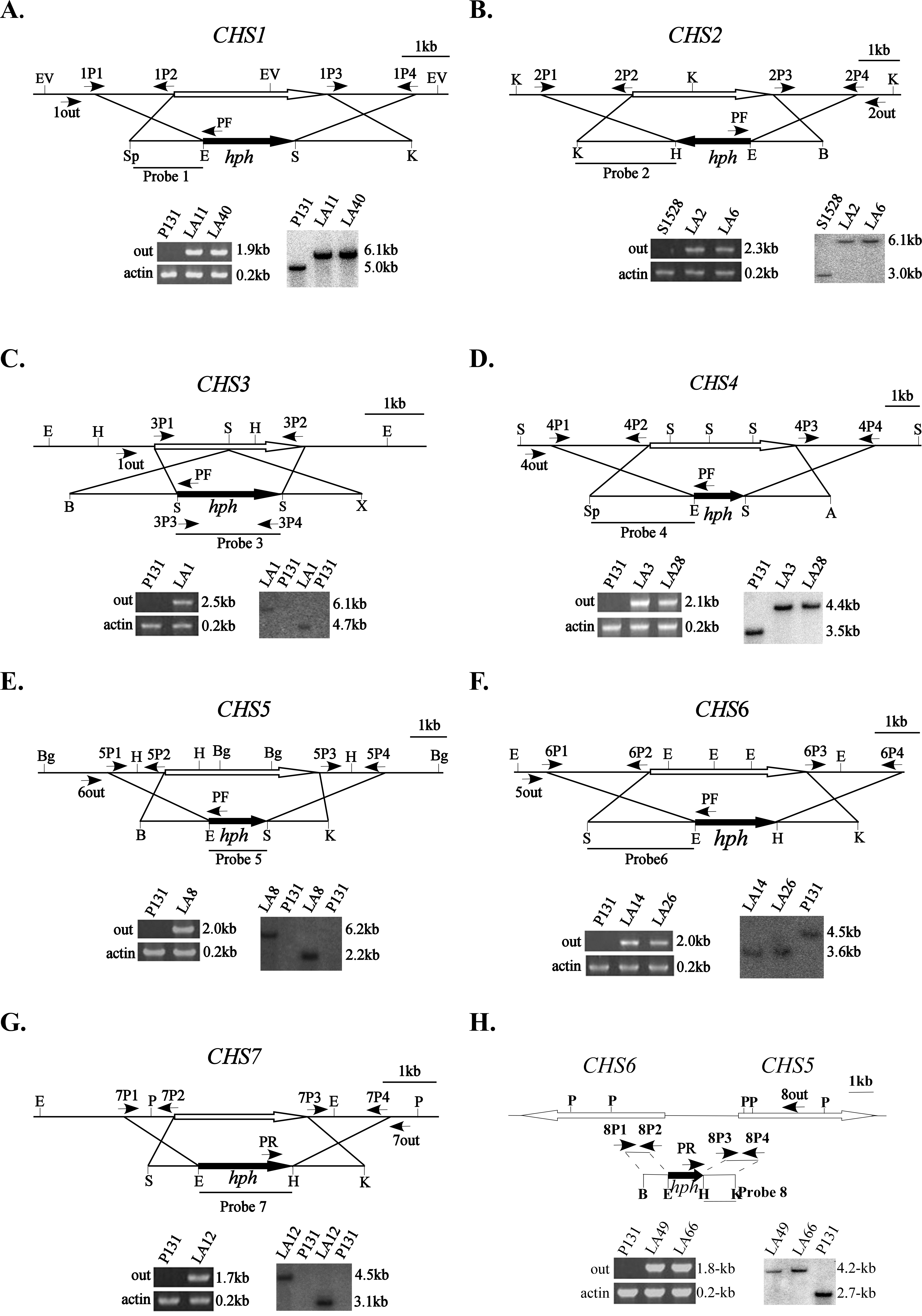

Supplement: Figure S2 — CHS gene deletion construct and confirmation. (A) CHS1 gene deletion strategy and confirmation. CHS1 deletion construct (the upper) was created by replacing the CHS1 gene with hygromycin phosphotransferase cassette (hph). The upstream and downstream flanking sequences were amplified with primers 1P1/1P2 and 1P3/1P4, and ligated with the hph cassette. The positions of primers 1P1, 1P2, 1P3, 1P4, 1out, and PF are indicated with small arrows. E, EcoRI; EV, EcoRV; K, KpnI; S, SalI; Sp, SpeI. This construct was introduced into the wild-type strain P131. The putative mutants were screened with primers 1out/PF, and the putative mutants LA11 and LA40 had 1.9-kb specific band. 0.2-kb fragment within MoACT1 gene was amplified as the endogenous reference (left bottom). Southern blot of EcoRV-digested genomic DNA of wild-type P131 and chs1 mutant LA11 and LA40 hybridized with Probe 1, which was amplified with primers 1P1/1P2. The results show a single 5.0-kb band (lane1) for the wild-type P131 and a single 6.0-kb band (lane 2 and lane 3) for chs1 mutant LA11 and LA40 (right bottom). (B) CHS2 gene deletion strategy and confirmation. CHS2 deletion construct (the upper) was created by replacing the CHS2 gene with hph. The upstream and downstream flanking sequences were amplified with primers 2P1/2P2 and 2P3/2P4, and ligated with the hph cassette. The positions of primers 2P1, 2P2, 2P3, 2P4, 2out, and PF are indicated with small arrows. B, BamHI; E, EcoRI; H, HindIII; K, KpnI. This construct was introduced into the wild-type S1528. The deletion mutants were screened with primers 2out/PF, and the putative mutants LA2 and LA6 had 2.3-kb specific band. 0.2-kb fragment within MoACT1 gene was amplified as the endogenous reference (left bottom). Southern blot of KpnI-digested genomic DNA of wild-type strain S1528 and chs2 mutant LA2 and LA6 hybridized with Probe 2, which was amplified with primers 2P1/1P2. The results show a single 3.0-kb band (lane 1) for the wild-type S1528 and a singl [file ppat.1002526.s002.tif]

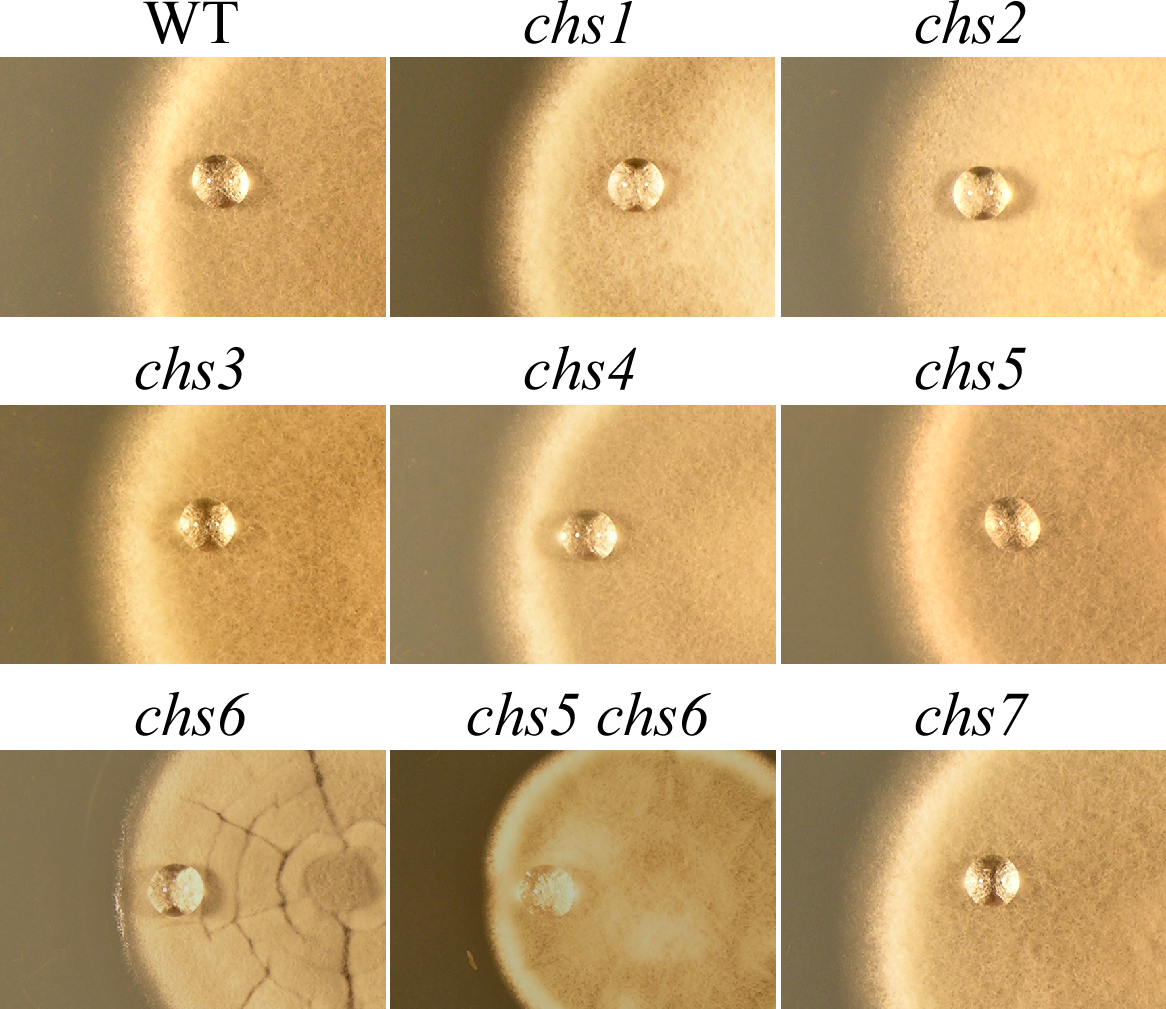

Supplement: Figure S3 — Hydrophobicity assay. Drops of 20 µl sterile distilled water were placed on the surface of vegetative colony from 7-day-old complement media agar cultures. The results show the surface of vegetative colony from the wild-type P131 and S1528, and seven CHS gene deletion mutants were hydrophobic. (TIF) [file ppat.1002526.s003.tif]

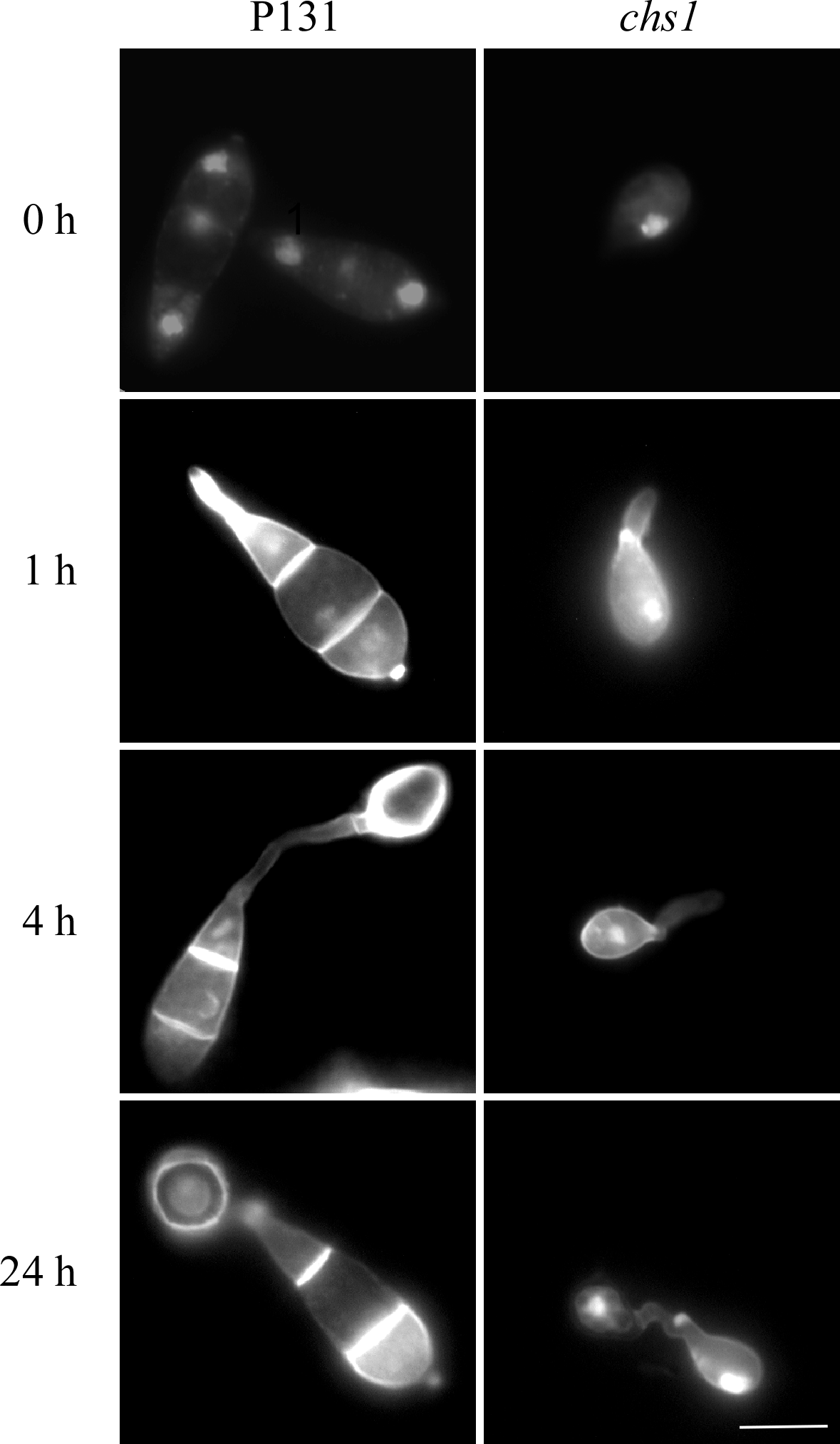

Supplement: Figure S4 — Nuclear division and movement during appressorium formation in the chs1 mutant. Conidia of the wild type and chs1 mutant incubated on hydrophobic glass coverslips for 0, 1, 4, and 24 h were stained with CFW and DAPI and examined with an epifluorescence microscope. Bar = 10 mm. (TIF) [file ppat.1002526.s004.tif]

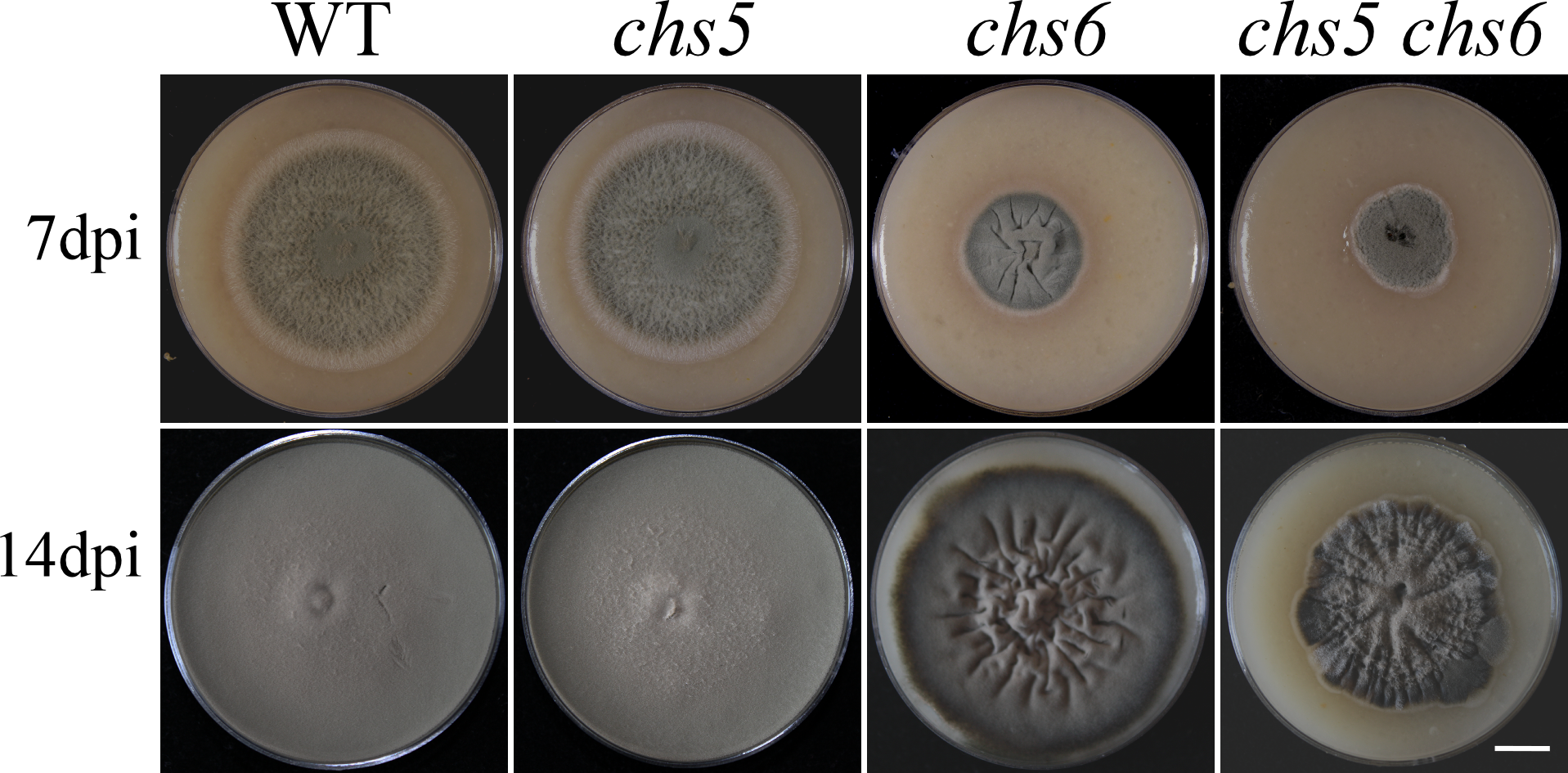

Supplement: Figure S5 — The chs5 chs6 double mutant was more significantly reduced in growth rate than chs6 mutant. The wild-type P131, chs5, chs6, and chs5 chs6 double mutant were cultured on OTA plates under light at 25°C. Photographs were taken at 7 and 14 dpi, respectively. Bar = 10 mm. (TIF) [file ppat.1002526.s005.tif]

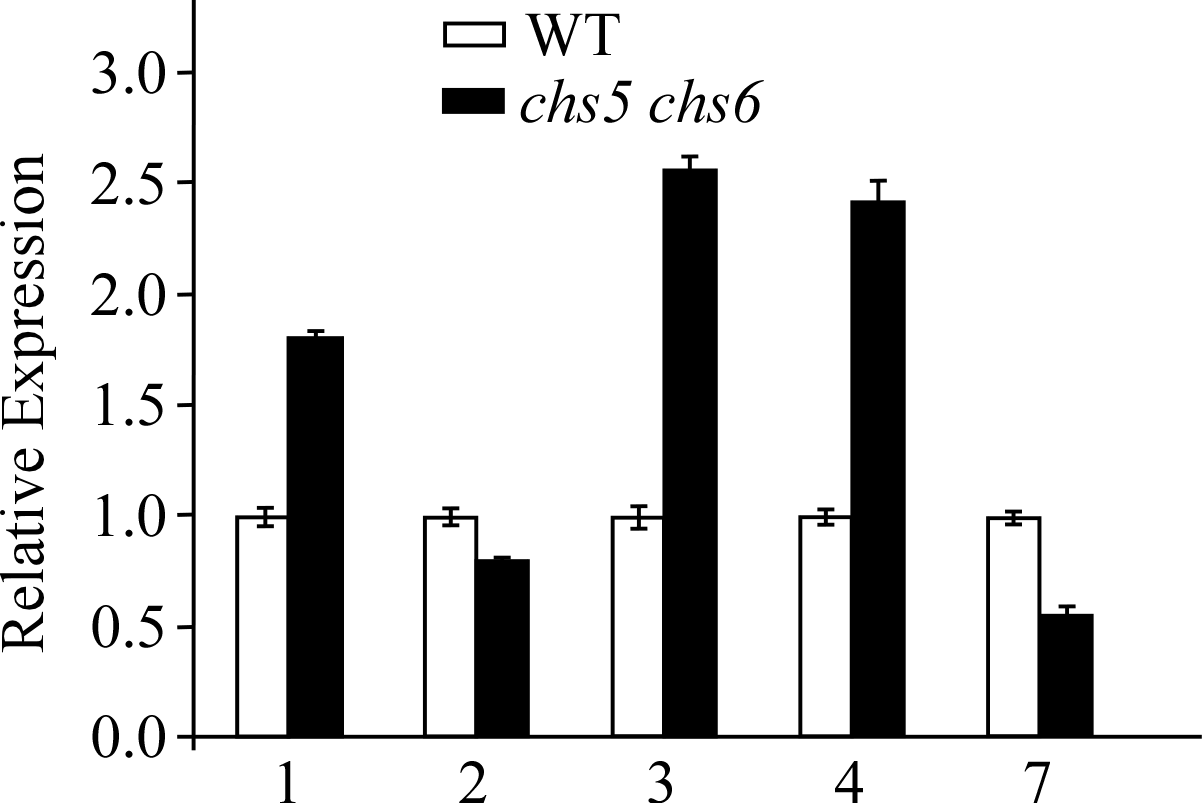

Supplement: Figure S6 — qRT-assays with the expression levels of CHS genes in the wild-type P131 and chs5 chs6 mutants. The RNA was isolated from vegetative mycelia shaken in CM for two days. The actin gene was used as the endogenous control for normalization. Relative expression levels were estimated with the 2−ΔΔCt method. The expression level of each CHS gene in the wild type was arbitrarily set to 1. Mean and standard errors were determined with data from three independent replicates. (TIF) [file ppat.1002526.s006.tif]
