# Supplementary material for: Different Chitin Synthase Genes Are Required for Various Developmental and Plant Infection Processes in the Rice Blast Fungus Magnaporthe oryzae
Source: PLoS Pathog. 2012 Feb 9;8(2):e1002526. doi: 10.1371/journal.ppat.1002526 (PMC3276572; doi:10.1371/journal.ppat.1002526)
Supplement: Table S1 — PCR primers used in this study. (DOC) [file ppat.1002526.s007.doc]

**Table S1. PCR primers used in this study.**

| **Primer** | **Sequence (5΄→3΄)** | **Application** |
| --- | --- | --- |
| 1P1 | tactagtcgatgtccgtcagcg | *chs1* deletion |
| 1P2 | agaattcctgagtgagatggcg | *chs1* deletion |
| 1P3 | agtcgacgactaaggcttggtg | *chs1* deletion |
| 1P4 | tggtaccaggacaccttcttcg | *chs1* deletion |
| 1out | ttgcgggtcaagcgttctg | *chs1* deletion |
| PF | gacagacgtcgcggtgagtt | hygup out |
| actinF | gaccgactacctgatgaaga | internal reference |
| actinR | tgccgatggtgataacctg | internal reference |
| 2P1 | tggtaccaacattcctagtcgg | *chs2* deletion |
| 2P2 | taagcttctcgtccactgcatc | *chs2* deletion |
| 2P3 | cgaattcggttcaggagttcag | *chs2* deletion |
| 2P4 | tggatcccacttcactctcttc | *chs2* deletion |
| 2out | aggtctacgcttgctcgcag | *chs2* deletion |
| 3P1 | tccacgacccaggatccgc | *chs3* deletion |
| 3P2 | cacatgggctcgaggtcgaa | *chs3* deletion |
| 3P3 | attgtcgacggcttggctggagctagtggaggtcaa | *chs3* deletion |
| 3P4 | attgtcgacgaacccgcggtcggcatctactctat | *chs3* deletion |
| 3out | cgaggtcacattgcgaatcaac | *chs3* deletion |
| 4P1 | tactagtcttgcgcaatctgtc | *chs4* deletion |
| 4P2 | ggaattcaagggtaggtgaatc | *chs4* deletion |
| 4P3 | tgtcgacaggcgaaggcctttg | *chs4* deletion |
| 4P4 | cgggccctgattttgaattgag | *chs4* deletion |
| 4out | atcgatcgtacaacccacgg | *chs4* deletion |
| 5P1 | tggatcctcgaacaattgcatc | *chs5* deletion |
| 5P2 | tgaattcccgtggctactgtacc | *chs5* deletion |
| 5P3 | cgtcgaccaaatgtctctgagc | *chs5* deletion |
| 5P4 | tggtacccatcgtccaagtctg | *chs5* deletion |
| 5out | ccattactgaggccaggattg | *chs5* deletion |
| 6P1 | gactagtccttgaccgagtgtcc | *chs6* deletion |
| 6P2 | tgaattcatgtgcctggagac | *chs6* deletion |
| 6P3 | actaagcttgcaaggcttcgtatg | *chs6* deletion |
| 6P4 | ataggtaccgtgtcggcatcgatc | *chs6* deletion |
| 6out | acgaaagtgggctggctctatc | *chs6* deletion |
| 7P1 | ggactagtcgctcctctatgaag | *chs7* deletion |
| 7P2 | cacgaattcacctgcctctaatgc | *chs7* deletion |
| 7P3 | cctaagcttaccaacctcctcaca | *chs7* deletion |
| 7P4 | tccggtacccaatacgataagaga | *chs7* deletion |
| 7out | cgtaacacaccaggcaca | *chs7* deletion |
| 8P1 | aacgaattctaccgaccagagtc | *chs5 chs6* deletion |
| 8P2 | gtaggatccgatctgaagcatg | *chs5 chs6* deletion |
| 8P3 | ctcaagcttggtgacaccctc | *chs5 chs6* deletion |
| 8P4 | cttggtaccagcaattggtctcg | *chs5 chs6* deletion |
| 8out | ttgaacaatggaggagggatg | *chs5 chs6* deletion |
| PR | tctggaccgatggctgtgtag | hygdown out |
| DW1F | cgtatcgatagccaccactgccttgttgtt | *CHS1-*eGFP |
| DW1R | ttaaactgatgcgacgggcaatgcagcacat | *CHS1-*eGFP |
| DW7F | atagggccccacatgttaatctcgctttgt | *CHS7-*eGFP |
| DW7R | aatatcgatttgctgatggccgccgctgtt | *CHS7-*eGFP |
| Ps | gccggtggtgcagatgaacttc | GFP sequence |
| actinQF | ccatgtaccctggtctttcg | qRT |
| actinQR | ttcgagatccacatctgctg | qRT |
| 1QF | tcaacgacgaggagaagcc | *CHS1* qRT |
| 1QR | gtaatcgcaacagccaaga | *CHS1* qRT |
| 2QF | tccacgacctttgccatca | *CHS2* qRT |
| 2QR | cgcttttgcttccgcgact | *CHS2* qRT |
| 3QF | cggaaaccaaggaacagcg | *CHS3* qRT |
| 3QR | cagggaacaaccaagaaccac | *CHS3* qRT |
| 4QF | tcgagggaaaatgtaacgg | *CHS4* qRT |
| 4QR | tactgctgctggtgatggt | *CHS4* qRT |
| 5QF | ccgtgttgatggaggttga | *CHS5* qRT |
| 5QR | gatctggcggtcgaggaat | *CHS5* qRT |
| 6QF | gaacggcagatttgatgac | *CHS6* qRT |
| 6QR | acaagagtgcttcggtggc | *CHS6* qRT |
| 7QF | gacattgagctggagattgg | *CHS7* qRT |
| 7QR | cgccgctgttgctgttgtt | *CHS7* qRT |
