# Supplementary material for: Different Chitin Synthase Genes Are Required for Various Developmental and Plant Infection Processes in the Rice Blast Fungus Magnaporthe oryzae
Source: PLoS Pathog. 2012 Feb 9;8(2):e1002526. doi: 10.1371/journal.ppat.1002526 (PMC3276572; doi:10.1371/journal.ppat.1002526)
Supplement: Table S2 — The effects of cutin monomer treatment on appressorium formation by the wild type and chs7 mutant on different surfaces. (DOC) [file ppat.1002526.s008.doc]

**Table S2. The effects of cutin monomer treatment on appressorium formation by the wild type and *chs7* mutant on different surfaces.**

| **% Appressoria by germ tubes** | | | | **% Appressoria by hyphal tips** | |
| --- | --- | --- | --- | --- | --- |
|  | Hydro- | Rice sheath^b^ | Barley | Hydro- | Hydro- |
|  | phobic(+)^a^ |  |  | phobic(-) | phobic(+) |
| P131 (WT) | 98.5±1.4 | 94.2±1.1 | 97.2±0.5 | 54.0±1.5 | 56.7±2.2 |
| LA12 (*chs7*) | 81.2±1.8 | 78.3±2.2 | 83.2±3.1 | 48.2±2.3 | 50.0±2.5 |

^a^The appressoria formation were assayed by germ tubes and hyphal tips on artificial hydrophobic surface of Gelbond film without (-) or within (+) 10 μM cutin mononer 1,16-hexadecanediol. ^b^The appressoria assayed was peroformed by germ tubes on rice leaf sheath and barley leaf surface. The formed appressoria by germ tubes and hyphal tips were counted at 24 and 48 hpi, respectively. The appressorial formation was measured as the ratio of appressoria-forming germ tubes or hyphal tips to the total germ tubes or hyphal tips. Means and standard deviations were calculated from three independent replicate experiments.
