# Supplementary material for: Different Chitin Synthase Genes Are Required for Various Developmental and Plant Infection Processes in the Rice Blast Fungus Magnaporthe oryzae
Source: PLoS Pathog. 2012 Feb 9;8(2):e1002526. doi: 10.1371/journal.ppat.1002526 (PMC3276572; doi:10.1371/journal.ppat.1002526)
Supplement: Table S3 — Defect of the chs 5 chs 6 mutants in vegetative growth. (DOC) [file ppat.1002526.s009.doc]

**Table S3. Defect of the *chs*5 *chs*6 mutants in vegetative growth.**

|  | **Colony diameter (mm)** ^*^ |
| --- | --- |
| P131 (WT) | 38.7±0.5**^α^** |
| LA8 (*chs5*) | 38.0±0.5**^α^** |
| LA26 (*chs6*) | 22.3±0.5**^β^** |
| LA49 (*chs5 chs6*) | 16.7±0.5**^γ^** |

^*^Colony diameters of oatmeal agar cultures were measured after incubation for 7 days.

Data from three replicates were analyzed with two sample t-test. The same greek letter indicated that there was no significant difference with the wild-type or single mutant. Different letters were used to mark statically significant difference (P=0.01).
